# Revision of the carnivorous snail genus *Discartemon* Pfeiffer, 1856, with description of twelve new species (Pulmonata, Streptaxidae)

**DOI:** 10.3897/zookeys.401.7075

**Published:** 2014-04-14

**Authors:** Thanit Siriboon, Chirasak Sutcharit, Fred Naggs, Ben Rowson, Somsak Panha

**Affiliations:** 1 Animal Systematics Research Unit, Department of Biology, Faculty of Science, Chulalongkorn University, Bangkok 10330, Thailand; 2 Biological Sciences Program, Faculty of Science, Chulalongkorn University, Bangkok 10330, Thailand; 3 Department of Life Sciences, The Natural History Museum, Cromwell Road, London SW7 5BD, United Kingdom; 4 Department of Natural Sciences, National Museum of Wales, Cathays Park, Cardiff CF10 3NP, United Kingdom

**Keywords:** Systematics, genitalia, predator, anatomy, Southeast Asia

## Abstract

Twelve new species of the streptaxid snail genus *Discartemon* Pfeiffer, 1856 are described from southern Thailand and western Malaysia, *D. afthonodontia* **sp. n.**, *D. circulus* **sp. n.**, *D. deprima* **sp. n.**, *D. discadentus* **sp. n.**, *D. discamaximus* **sp. n.**, *D. expandus* **sp. n.**, *D. flavacandida* **sp. n.**, *D. kotanensis* **sp. n.**, and *D. megalostraka* **sp. n.** from southern Thailand, as well as *D. conicus* **sp. n.**, *D. epipedis* **sp. n.** and *D. triancus* **sp. n.** from western Malaysia. All 15 previously described species are revised and commented upon based on examined material. *Streptaxis paradiscus* Möllendorff, 1900 is considered a junior subjective synonym of the type species *D. discus* (Pfeiffer, 1853). Details of the genital anatomy of twelve species, and the radula and pallial system, are provided for the first time. An identification key is provided.

## Introduction

The Streptaxoidea is divided into two sister families, Streptaxidae Gray, 1860 and Diapheridae Panha & Naggs, 2010 (Sutcharit et al. 2010). The superfamily is thought to have originated on the Laurasian continent during the Mesozoic era (Rowson et al. 2010). The Streptaxidae are carnivorous land snails occurring in tropical and sub-tropical areas from South America to Africa and Asia (Bruggen 1967, Schileyko 2000, Sutcharit et al. 2010). Most appear to be active predators feeding on other snails or other soil invertebrates, and may also be cannibalistic (Gray 1860, Blanford and Godwin-Austen 1908, Benthem Jutting 1954, Berry 1963). Streptaxids are particularly diverse in Africa, with hundreds of described species (Bruggen 1967, Winter and Gittenberger 1998, Rowson et al. 2010, Rowson and Tattersfield 2013). They are also diverse in Southeast Asia, comprising more than 130 nominal species in 15 genera (Blanford and Godwin-Austen 1908, Benthem Jutting 1954, Zilch 1960, Richardson 1988, Schileyko 2000, Siriboon et al. 2013, Siriboon et al. in press).

The shell has traditionally been emphasized in streptaxid taxonomy (Tryon 1885, Kobelt 1905, 1906, Benthem Jutting 1954). As in many stylommatophorans, the reproductive organs have also proven to be useful in discriminating taxa at the generic and specific levels (Stoliczka 1871, Berry 1963, 1965, Schileyko 2000, Siriboon et al. 2013, in press). To date, six generally accepted Southeast Asian genera, *Discartemon* Pfeiffer, 1856, *Oophana* Ancey, 1884, *Perrottetia* Kobelt, 1905, *Haploptychius* Möllendorff, 1906 and *Indoartemon* Forcart, 1946, have been critically dissected, investigated and illustrated, providing additional anatomical diagnostic and systematic characters (Stoliczka 1871, Berry 1963, 1965, Schileyko 2000, Siriboon et al. 2013, in press).

The genus *Discartemon* can be distinguished from other Southeast Asian streptaxid genera by having a flattened to subglobose-heliciform shell with the last whorl not being axially distorted from the columellar axis (Kobelt 1906, Benthem Jutting 1954, Zilch 1960, Richardson 1988, Schileyko 2000). *Discartemon* species are all larger than those of *Platycochlium* Laidlaw, 1950, a genus from Borneo whose anatomy is not known, and do not share the riblets on slopes of umbilicus, spaced transverse ridges, and continuous peristome. Knowledge of the genital anatomy of *Discartemon* is currently limited to *Discartemon stenostomus* Benthem Jutting, 1954 as studied by Berry (1965). The genitalia show a short penis with a blunt appendix and a penial sheath along its whole length. The internal penial wall has cornified ridges but no penial hooks apart from a single large “stylet” in the apex of the penis.

The genus currently includes 15 nominal species and ranges from the Isthmus of Kra to peninsular Malaysia, with a few species recorded from Sumatra, Sulawesi and Indochina (Benthem Jutting 1954, 1959, Bruggen 1967, 1972, Richardson 1988). Most species have narrow distributions. Ten are recorded in peninsular Malaysia (Benthem Jutting 1954, 1959, Maassen 2001). Four species, *Discartemon roebeleni* (Möllendorff, 1894), *Discartemon sykesi* (Collinge, 1902), *Discartemon nummus* (Laidlaw, 1929) and *Discartemon khaosokensis* Panha & Burch, 1998 have previously been recorded from Thailand (Panha 1996, Hemmen and Hemmen 2001). Two species occur in Cambodia and Vietnam, two further ones were recorded from Sumatra and one species was described from Sulawesi (Morlet 1889, Kobelt 1906, Laidlaw 1933, Benthem Jutting 1959, Marwoto 2008, Schileyko 2011). The Sulawesi species of *Discartemon* represents one of only two streptaxid genera recorded from Sulawesi, the other being *Haploptychius*, of which three species are recorded (Bruggen 1972).

This present study aims firstly to provide shell and anatomical descriptions for characterization and identification within the genus *Discartemon*, including new species The second aim is to revise the previously described species. The third aim is to record and discuss the geographic distribution of the genus.

## Material and methods

Streptaxids were intensively surveyed throughout southern Thailand and western Malaysia and Vietnam from 1995–2012. Identifications were provisionally made based on Kobelt (1906) and Benthem Jutting (1954, 1959) and comparison with type specimens from many museums. Living snails were photographed before being stored at -20 °C prior to being preserved in 70% and 95% ethanol for anatomical and molecular studies. Shell height (H), shell width (W), whorl counts and H/W ratio were measured and calculated. Shells were digitally imaged using Cell’D Imaging Software. Description of apertural dentition follows Pilsbry (1916) and Siriboon et al. (2013). The genitalia of 5–10 specimens of each species were dissected under a stereo-microscope. Anatomical sketches were drawn using a camera lucida. The buccal masses were removed, and the radulae were soaked in 10% sodium hydroxide, cleaned in distilled water, examined and photographed under SEM (JEOL, JSM-5410 LV). Atrial and penial and vaginal hooks were critical point dried by using absolute ethanol prior to investigation under SEM (PHILIPS, XL30). In the descriptions, ‘proximal’ relates to the genital orifice, and ‘distal’ to the region furthest away from the genital orifice. Apart from the term ‘penial appendix’ terms are as defined by Stoliczka (1871), Berry (1963), Verdcourt (2000), Sutcharit et al. (2010) and Siriboon et al. (2013)

**Anatomical abbreviations:** a, anus; ag, albumen gland; at, atrium; fo, free oviduct; gd, gametolytic duct; gs, gametolytic sac; h, heart (auricles and ventricle); hd, hermaphroditic duct; k, kidney; ov, oviduct; p, penis; pa, penial appendix; pn, pneumostome; pp, penial papilla; pr, penial retractor muscle; ps, penial sheath; psr, penial sheath retractor muscle; puv, pulmonary vein; rt, rectum; sv, seminal vesicle; ta, talon; ur, ureter; v, vagina; vd, vas deferens.

**Institutional abbreviations:** Examined material was deposited in the following institutions:

CUMZ Chulalongkorn University Museum of Zoology, Bangkok;

MNHN Muséum National ďHistoire Naturelle, Paris;

NHMUK The Natural History Museum, London;

NHMW Naturhistorishes Museum Wien, Vienna;

NMW National Museum of Wales, Cardiff;

RMNH National Museum of Natural History Naturalis, Leiden;

RBINS Royal Belgian Institute of Natural Sciences, Brussels;

SMF Forschungsinstitut und Naturmuseum Senckenberg, Frankfurt am Main;

ZMA Zoological Museum Amsterdam, Amsterdam;

ZMB Museum für Naturkunde, Berlin.

All descriptions of the new species are here attributed to the first and the fourth author, Siriboon and Panha, respectively.

## Systematics

### Family Streptaxidae Gray, 1860

#### Genus Discartemon

Pfeiffer, 1856

http://species-id.net/wiki/Discartemon

Discartemon Pfeiffer, 1856: 173. Ancey 1884: 399. Tryon 1885: 58. Gude 1903: 226. Benthem Jutting: 1954: 71–94. Zilch 1960: 560. Richardson 1988: 182–185: Schileyko 2000: 784. Hemmen and Hemmen 2001: 42.
Odontartemon (Discartemon) – Kobelt 1905–1906: 91, 96.

##### Type species.

*Streptaxis discus* Pfeiffer, 1853, by subsequent designation by Ancey (1884: 399).

##### Description.

**Shell.** Shell flattened to globose-heliciform, white, semi-transparent to translucent. Whorls 4–7; spire flattened to conical. Shell surface glossy, nearly smooth or with transverse ridges; varices often present. Embryonic shell, about 2½ whorls, with a smooth surface; following whorls regularly coiled or at most only slightly axially deflected. Last whorl rounded to angular, often with peripheral keel, whorls regularly to rapidly expanded. Umbilicus open to very widely open. Aperture semi-ovate to triangular. Peristome discontinuous, thin to thick, expanded and reflected. Longitudinal furrows outside aperture may be present. Apertural dentition always with one parietal lamella; other lamellae may be present including: upper palatal, palatal, basal, columellar and supracolumellar lamellae.

**Radula.** Teeth unicuspid, elongate lanceolate, and arranged in anteriorly V-shaped rows. Central tooth tiny with pointed cusp. Lateral and marginal teeth undifferentiated. Latero-marginal teeth gradually reduce in size, with outermost teeth smaller and shorter than inner teeth.

**Genital organs.** Penis short to long, sometimes with a penial appendix. Penial sheath short (less than half of penis length) to long (equivalent to penis length). Internal wall of introverted penis with transparent to brown penial hooks. Vas deferens passes through a short section of penial sheath before connecting distally to penis. Vagina and free oviduct short to long. Seminal vesicle present, convoluted, short to long.

**External features.** Live specimens exhibit a semi-transparent dark yellow to pale yellow body, covered with reticulated skin, and sometimes with brownish spots. Upper tentacles long with black eye-spot on the tip, yellow to orange; lower tentacles short. Brownish digestive gland and black kidney may be visible through transparent shell. Foot narrow, undivided and with short tail.

##### Remarks.

The genitalia of *Discartemon* are distinguished from those of other Southeast Asian streptaxid genera in sometimes having a penial appendix, in lacking vaginal hooks, and also as follows: *Indoartemon* has the vas deferens attached to the distal end of the penial sheath by a narrow band of connective tissue; in *Perrottetia* the gametolytic duct and sac may not extend as far as the albumin gland; and *Haploptychius* and *Oophana* have a long penial sheath and very short seminal vesicle respectively (Stoliczka 1871, Berry 1963, 1965, Schileyko 2000, Siriboon et al. 2013, Siriboon et al. in press).

**Figure 1.**
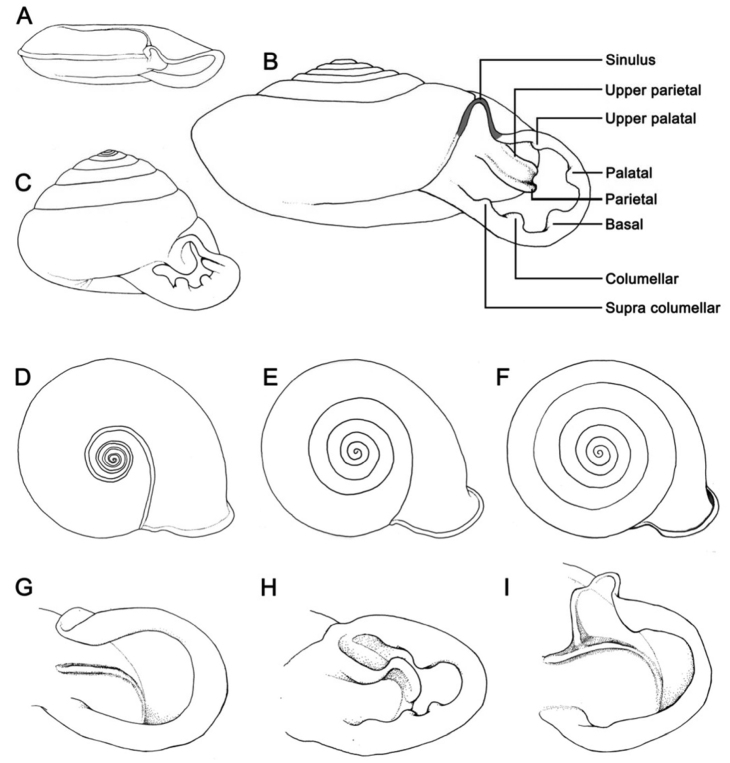
Schematic of shell shapes, last whorl expansion and parietal lamella shape. Terminology of *Discartemon* apertural dentition in figure **B. A–C** Shell form and spire **A** flattened shell with concave spire **B** depressed-heliciform shell with only slightly convex spire, and **C** globose-heliciform with conical spire **D–F** Last whorl expansion **D** rapidly expanded **E** intermediately expanded, and **F** regularly expanded **G–I** Parietal lamella form **G** single lamella with straight shape (typical) **H** single lamella with curved shape (sinuous), and **I** modified with “Y” shaped lamella.

An identification key to species follows. In addition we propose an informal subdivision of *Discartemon* into three groups of species, based mainly on shell shapes as shown in Figure 1, that may be useful as an alternative aid to identification. The figures of shells are presented in the same order.

Further remarks on the systematics and biogeography of the genus are made in the Discussion.

Group I: *Discartemon discus*-group. Have a generally flattened shell with a concave to flattened spire, and a very wide umbilicus. The H/W ratio ranges between 0.3–0.5 (average 0.40). This group comprises 10 species: *Discartemon discus* (Pfeiffer, 1853), *Discartemon planus* (Fulton, 1899), *Discartemon sykesi* (Collinge, 1902), *Discartemon nummus* (Laidlaw, 1929), *Discartemon khaosokensis* Panha & Burch, 1998, *Discartemon circulus* sp. n., *Discartemon discadentus* sp. n., *Discartemon discamaximus* sp. n., *Discartemon deprima* sp. n., and *Discartemon expandus* sp. n.

Group II: *Discartemon plussensis*-group. Have a depressed-heliciform shell with a flattened to only slightly convex spire, and a widely open umbilicus. The H/W ratio ranges between 0.4–0.6 (average 0.50). This group comprises 7 species: *Discartemon plussensis* (Morgan, 1885), *Discartemon hypocrites* Benthem Jutting, 1954, *Discartemon leptoglyphus* Benthem Jutting, 1954, *Discartemon platymorphus* Benthem Jutting, 1954, *Discartemon afthonodontia* sp. n., *Discartemon epipedis* sp. n., and *Discartemon flavacandida* sp. n.

Group III: *Discartemon roebeleni*-group. Have a globose-heliciform shell with a conical to elevated conical spire, and a widely open umbilicus. The H/W ratio ranges between 0.5–0.8 (average 0.63). This group comprises 10 species: *Discartemon lemyrei* (Morlet, 1883), *Discartemon roebeleni* (Möllendorff, 1894), *Discartemon collingei* (Sykes, 1902), *Discartemon stenostomus* Benthem Jutting, 1954, *Discartemon sangkarensis* Benthem Jutting, 1959, *Discartemon vandermeermohri* Benthem Jutting, 1959, *Discartemon conicus* sp. n., *Discartemon kotanensis* sp. n., *Discartemon megalostraka* sp. n., and *Discartemon triancus* sp. n.

#### Key to species of *Discartemon* Pfeiffer, 1856

Numbers for each species refer to the order in which species treatments appear in this paper.

**Table:** 

1a	Shell flattened (Fig. 1A); spire concave, flattened or only slightly elevated	2
1b	Shell depressed (Fig. 1B) - or globose-heliciform (Fig. 1C); spire flattened, convex or conical	11
2a	Shell width usually greater than 10 mm	3
2b	Shell width usually less than 10 mm	6
3a	Spire concave; last whorl angular with strong peripheral keel	5. *Discartemon khaosokensis*
3b	Spire flattened or only slightly elevated; last whorl angular	4
4a	Apertural dentition with four or five lamellae: parietal, palatal, basal, columellar and small supracolumellar lamellae (the last sometimes absent)	6. *Discartemon discadentus* sp. n.
4b	Apertural dentition with only a parietal lamella (Fig. 1G)	5
5a	Last whorl regularly expanded (Fig. 1F); shell surface smooth; spire flattened	1. *Discartemon discus* (= *Discartemon paradiscus*)
5b	Last whorl rapidly expanded (Fig. 1D); transverse ridges present only near suture; spire flattened to concave	7. *Discartemon discamaximus* sp. n.
6a	Spire concave	7
6b	Spire flattened	8
7a	Last whorl rounded, rapidly expanded; aperture triangular. Apertural dentition with parietal, palatal and columellar lamellae	2. *Discartemon planus*
7b	Last whorl angular with strong peripheral keel, intermediately expanded (Fig. 1E); aperture semi-ovate. Apertural dentition with only a parietal lamella	9. *Discartemon deprima* sp. n.
8a	Apertural dentition with five lamellae: parietal, palatal, basal, columellar and supracolumella lamellae	8. *Discartemon circulus* sp. n.
8b	Apertural dentition with one or two lamellae: parietal and columellar lamellae	9
9a	Shell width usually less than 7 mm; last whorl angular with peripheral keel; Y-shaped parietal lamella (Fig. 1I)	4. *Discartemon nummus*
9b	Shell width greater than 7 mm; last whorl angular or rounded; straight parietal lamella	10
10a	Shell surface smooth; peristome thickened and expanded	3. *Discartemon sykesi*
10b	Shell surface with transverse ridges disappearing below periphery; peristome thin and widely expanded	10. *Discartemon expandus* sp. n.
11a	Shell depressed-heliciform; spire flattened to convex (Fig. 1B)	12
11b	Shell globose-heliciform; spire conical to elevated conical (Fig. 1C)	18
12a	Shell surface usually smooth or with few transverse ridges near aperture. Last whorl shouldered or angular and with strong peripheral keel	13
12b	Shell surface with fine transverse ridges. Last whorl angular or rounded	15
13a	Longitudinal furrow absent. Apertural dentition with four lamellae: parietal, palatal, basal and columellar lamellae	16. *Discartemon epipedis* sp. n.
13b	Two longitudinal furrows present. Apertural dentition with five to seven lamellae	14
14a	Shell width usually greater than 10 mm; spire only slightly convex; varices absent. Last whorl slightly axially deflected. Apertural dentition with seven lamellae: parietal, upper parietal, upper palatal, palatal, basal, columellar and supracolumellar lamellae. The two latter lamellae usually small. Penis long, about same length as free oviduct; penial appendix present	17. *Discartemon flavacandida* sp. n.
14b	Shell width usually less than 10 mm; spire conical; varices present. Last whorl regularly coiled. Apertural dentition usually with five lamellae: parietal, palatal, basal, columellar and supracolumellar lamellae. An additional upper parietal and upper palatal lamellae are sometimes present. Penis short, about ¼ length of free oviduct; penial appendix absent	15. *Discartemon afthonodontia* sp. n.
15a	Apertural dentition with four lamellae: sinuous parietal (Fig. 1H), palatal, columellar and supracolumellar lamellae	12. *Discartemon hypocrites*
15b	Apertural dentition with only one lamella or two lamellae: parietal and palatal lamellae	16
16a	Transverse ridges present over entire shell; last whorl angular and less inflated	13. *Discartemon leptoglyphus*
16b	Transverse ridges disappear below periphery; last whorl rounded and more inflated	17
17a	Shell width usually less than 7 mm; spire flattened; sinulus present	11. *Discartemon plussensis*
17b	Shell medium (7 ≤ shell width ≤ 10 mm); spire low convex; sinulus absent	14. *Discartemon platymorphus*
18a	Apertural dentition with only parietal lamella	19
18b	Apertural dentition with four lamellae	22
19a	Umbilicus usually narrow; sinulus absent	18. *Discartemon lemyrei*
19b	Umbilicus widely open; sinulus present	20
20a	Shell surface with fine transverse ridges; spire conical; last whorl rounded; aperture triangular	21
20b	Shell surface smooth; spire elevated conical; last whorl angular; aperture subcircular	27. *Discartemon conicus* sp. n.
21a	Apertural dentition with only parietal lamella; sinulus present	22. *Discartemon sangkarensis*
21b	Apertural dentition with two lamellae: parietal and columellar lamellae; sinulus absent	23. *Discartemon vandermeermohri*
22a	Spire only slightly convex; last whorl shouldered or angular	23
22b	Spire conical to elevated conical; last whorl rounded	24
23a	Parietal lamella sinuous; supracolumellar lamella present	21. *Discartemon stenostomus*
23b	Parietal lamella straight; basal lamella present	26. *Discartemon triancus* sp. n.
24a	Shell width usually greater than 10 mm. Free oviduct very long	25. *Discartemon megalostraka* sp. n.
24b	Shell width usually less than 10 mm. Free oviduct very short	25
25a	Last whorl shouldered and slightly axially deflected	20. *Discartemon collingei*
25b	Last whorl rounded and regularly coiled	26
26a	Spire elevated conical. Penis about four times longer than free oviduct and seminal vesicle short	24. *Discartemon kotanensis* sp. n.
26b	Spire conical. Penis about same length as vagina and free oviduct and seminal vesicle very long	19. *Discartemon roebeleni*

#### Group I: *Discartemon discus*-group: Species with flattened shell

##### 1. Discartemon discus

(Pfeiffer, 1853) [“1851”]

http://species-id.net/wiki/Discartemon_discus

Figs 4A–C , 11A–C , 22A , 23 , Table 1

Streptaxis discus Pfeiffer, 1851: 252. Type locality: Unknown. Pfeiffer 1853: 289. Pfeiffer 1854: 394, 395, pl. 145, figs 15–17. Ancey 1884: 399. Tryon 1885: 66, pl. 16, figs 77–79. Gude 1903: 226.
Discartemon discus – Bourguignat 1899: 46. Richardson 1988: 182. Schileyko 2000: 784, fig. 1022.
Streptaxis (Discartemon) paradiscus Möllendorff, 1900: 117. Type locality: Phucson bei Touranne, Annam. Gude 1903: 227. Ancey 1904: 289, 290.
Odontartemon (Discartemon) discus – Kobelt 1906: 97, pl. 55, figs 5–7.
Odontartemon (Discartemon) paradiscus – Kobelt 1906: 97, 98, pl. 55, figs 8, 9.
Discartemon paradiscus – Benthem Jutting 1954: 79. Zilch 1960: fig. 1961. Zilch 1961: 82, pl. 5, fig. 3. Schileyko 2011: 22, 23.

###### Material examined.

This species was described from specimens from the H. Cuming collection. The number of specimens was not indicated, but only one set of measurements was given in the original description. Only one specimen from the H. Cuming collection at NHMUK has Pfeiffer’s handwriting on the species name label. It is identical to the illustration and measurements in Pfeiffer (1854: 394, 395, pl. 145, figs 15–17) and is designated here as the lectotype to stabilize the name: NHMUK 20130684 (Fig. 4A).

Lectotype of *Streptaxis paradiscus* Möllendorff, 1900 SMF 108534 (Fig. 4B) and paralectotypes SMF 108535 (5 shells). Marble Mountain, Da Nang, Vietnam (16°0'13.4"N, 108°15'49.1"E): CUMZ 6001 (39 shells; Fig. 4C), 6257 (6 specimens in ethanol; Figs 11A, B, 22A). Annam: MNHN Jousseaume Coll. (1 shells), MNHN Denis Coll. (2 shells), MNHN Letellier Coll. (3 shells), NHMW 40858 (2 shells), NHMUK 1901.12.23.13–14 (2 shells), NHMUK Trechmann coll. Acc. 2176 (2 shells), NHMUK Connolly Coll. Acc. 2154 (1 shell), RMNH Fruhstorfer Coll. 45a (1 shell), RMNH Saverbgen Coll. (2 shells). Tourane [=Da Nang], Central Annam: NHMUK McAndrew coll. Acc. 1563 (2 shells). Touraine, Annam: NHMW Rusnov Coll. R 283 (2 shells), NMW 1955.158.25251 (1 shell), RMNH Verdcourt Coll. (2 shells), ZMB 6619 (3 shells), ZMB 52300 (3 shells). Phuc-Son, Annam: NHMW 31140 (1 shell).

###### Description.

**Shell.** Shell flattened, white and translucent; whorls 6–6½; spire flattened with distinct suture. Shell surface glossy, smooth with growth lines and varices present. Embryonic shell large, about 2½ whorls, with a smooth surface; following whorls regularly coiled. Last whorl angular, regularly expanded; umbilicus very wide, shallow and showing all preceding whorls. Aperture semi-ovate; peristome discontinuous, thickened, expanded and reflected; apertural dentition with only one parietal lamella (Fig. 4A–C).

**Radula.** Each row consists of 61–67 teeth with formula (30-33)-1-(30-33). Central tooth very small and triangular with pointed cusp. Lateral and marginal teeth undifferentiated, unicuspid and lanceolate. Latero-marginal teeth gradually reduce in size, with outermost teeth smaller and shorter than inner teeth (Fig. 22A).

**Genital organs.** Atrium (at) short; penis (p) long and slender. Penial sheath (ps) thin, extending about half to third-fourths of penis length; penial sheath retractor muscle (psr) very thin, originating at atrium and inserting at distal end of penial sheath (Fig. 11A). Vas deferens (vd) passes through a very short part of penial sheath before entering into penis distally (Fig. 11B). Penial retractor muscle (pr) thin and very long, inserting at penis and vas deferens junction.

Vagina (v) long, cylindrical, about two thirds of penis length. Gametolytic duct (gd) a long tube extending as far as albumin gland; gametolytic sac (gs) ovate. Free oviduct (fo) short; oviduct (ov) enlarged and folded. Prostate gland inconspicuous and bound to oviduct. Talon (ta) small. Hermaphroditic duct (hd) bearing long seminal vesicle (sv) about one and half times longer than the length from talon to branching point of seminal vesicle (Fig. 11A).

**Pallial system.** Excretory system typically sigmurethran and without mantle gland. Heart (h, auricles and ventricle) located left of kidney (on right in Fig. 11C). Pulmonary cavity approximately three times longer than broad. Pulmonary vein (puv) and venation on lung roof distinct and well developed. Kidney (k) very short, located at posterior of pulmonary cavity. Ureter (ur) sigmoid, closed tube arising from apex of kidney, extending along right side of kidney, recurving adjacent to rectum (rt). Anus (a) adjacent to pneumostome (pn) on mantle collar.

**Table 1.** Shell measurements of *Discartemon* spp (*Discartemon discus*-group). Specimen collections and catalogue numbers indicated in parentheses.

Species and locality and CUMZ nos	No. of specimens	Ranges, mean ± S.D. in mm of:			Number of whorls
		Shell height	Shell width	H/W ratio	
*Discartemon discus* (Pfeiffer, 1853)					
Da Nang, Vietnam: (6001, 6257)	45	4.2–6.7 5.0±0.43	11.78–14.26 12.9±0.59	0.3–0.5 0.4±0.03	6–6½
*Discartemon nummus* (Laidlaw, 1929)					
Khao Ok Thalu, Phatthalung: (3594)	24	2.4–3.3 2.8±0.21	6.1–7.2 6.5±0.27	0.4–0.5 0.4±0.03	5½
*Discartemon khaosokensis* Panha & Burch, 1998					
Khao Sok N. P., Suratthani: (6242, 6243)	5	3.4–4.0 3.6±0.26	11.2–12.4 11.8±0.47	0.3–0.3 0.3±0.02	5½–5¾
*Discartemon discadentus* sp. n.					
Wat Tam Yai, Suratthani: (6209, 6244, 6258)	16	3.9–5.8 5.0±0.49	10.1–15.4 11.9±0.32	0.4–0.5 0.4±0.04	6
Wat Tam Wararam, Suratthani: (3571)	15	5.5–7.0 6.3±0.33	12.4–13.6 13.1±0.32	0.4–0.5 0.4±0.02	6
*Discartemon discamaximus* sp. n.					
Tam Namphud, Phangnga: (6005, 6245)	5	4.7–5.0 4.9±0.10	12.4–13.6 14.3±0.49	0.3–0.4 0.3±0.02	7
Tam Kobe, Phangnga: (3669, 6197)	17	4.4–5.6 4.9±0.38	10.8–13.7 12.3±0.76	0.4–0.4 0.4±0.02	7
*Discartemon circulus* sp. n.					
Tam Phannara, Nakhon Si Thammarat: (3665, 6246, 6262)	23	3.0–4.5 3.7±0.32	7.7–9.5 8.6±0.45	0.4–0.5 0.4±0.03	5½–6
*Discartemon deprima* sp. n.					
Khao Hup Ta Hae, Prathiew, Chumphon: (3573, 6247)	7	2.5–3.4 2.9±0.30	8.2–10.3 9.1±0.72	0.3–0.3 0.3±0.02	5–5½
Ban Tam Thong, Prathiew, Chumphon: (6259)	5	3.1–3.8 3.5±0.27	9.5–11.2 10.1±0.68	0.3–0.3 0.3±0.01	5–5½
*Discartemon expandus* sp. n.					
Klong Hoy, Suratthani: (3664, 6248)	16	3.6–4.3 4.0±0.30	8.2–10.9 9.9±0.84	0.4–0.5 0.4±0.03	5½–6

###### Remarks.

The type specimen discovered in the H. Cuming collection at NHMUK elucidates two issues. Firstly, *Streptaxis paradiscus* Möllendorff, 1900 has been recognized as a separate species in many works (Möllendorff 1900, Gude 1903, Kobelt 1906, Benthem Jutting 1954, Zilch 1960, 1961, Schileyko 2011). However, based on the type specimens, *Discartemon discus* and *Streptaxis paradiscus* are identical in all shell characters. Therefore, we officially place *Streptaxis paradiscus* as a junior subjective synonym of *Discartemon discus*. Second, *Discartemon discus* had an unknown type locality and range (Pfeiffer 1851: 252). From the new material and the type locality of *Streptaxis paradiscus*, the distribution of this species is demarcated to several localities in the area of Da Nang, Vietnam (Schileyko 2011).

The record of *Streptaxis discus* from Brazil, mentioned in Bourguignat (1899) is almost certainly an error, since it is far beyond the distribution range of the genus. The specimen figured in Simone (2006: 191, fig. 708, reg. NHMUK Trechmann Acc. 2176) has the locality Annam [= central Vietnam].

##### 2. Discartemon planus

(Fulton, 1899)

http://species-id.net/wiki/Discartemon_planus

Figs 4D , 23

Streptaxis planus Fulton, 1899: 214, pl. 11, fig. 2. Type locality: South Celebes. Gude 1903: 227. Laidlaw 1933: 233. Sarasin and Sarasin 1899: 228.
Odontartemon (Discartemon) planus – Kobelt 1906: 100, 101, pl. 54, figs 15–17. Kobelt 1910: 150. Laidlaw 1929: 260.
Discartemon planus – Benthem Jutting 1954: 79. Bruggen 1972: 394. Richardson 1988: 183. Maassen 1997: 55. Marwoto 2008: 191–194, fig. 1.

###### Material examined.

Celebes [=Sulawesi], Indonesia: NMW 1955.158.25252 (1 shell; Fig. 4D).

###### Remarks.

The shell of this species is clearly distinct from all other recognized species. Shell flattened, with a concave spire and distinct suture. Shell surface smooth, varices present; whorls regularly coiled. Last whorl rounded with keel below periphery, rapidly expanded; umbilicus very wide, concave and showing all preceding whorls. Aperture triangular with long and narrow sinulus, peristome thickened and little reflected. Apertural dentition with one parietal, one palatal and one columellar lamella (Fig. 4D) (Fulton 1899, Kobelt 1906, Marwoto 2008).

The distribution of *Discartemon planus* seems to be outside the ranges of all other *Discartemon* species, and is probably restricted to the limestone karst in the south of Sulawesi (Fulton 1899, Sarasin and Sarasin 1899, Laidlaw 1929, Bruggen 1972, Marwoto 2008). It does not closely resemble any other streptaxid genus more closely than *Discartemon*. However, the very wide umbilicus showing all preceding whorls and surrounded with a keel, with a long and narrow adapical sinulus, may indicate that *Discartemon planus* comprises a distinct lineage within *Discartemon*. Both Bruggen (1972) and Marwoto (2008) discussed the possibility that it required a separate genus or subgenus, but anatomical or molecular evidence are desirable to support this assertion.

##### 3. Discartemon sykesi

(Collinge, 1902)

http://species-id.net/wiki/Discartemon_sykesi

Figs 4E, F , 23

Streptaxis sykesi Collinge, 1902: 72, pl. 4, figs 1, 2. Type locality: Biserat, State of Jalor. Laidlaw 1933: 233.
Odontartemon (Discartemon) sykesi – Kobelt 1906: 100, pl. 55, figs 1, 2. Kobelt 1910: 150.
Discartemon sykesi – Benthem Jutting 1954: 86, 87. Benthem Jutting 1959: 168. Richardson 1988: 184, 185. Maassen 2001: 88, 89. Hemmen and Hemmen 2001: 42.

###### Material examined.

Paratypes NHMUK 1937.7.9.11 (1 shell; Fig. 4F) and NMW 1955.158.25257 (1 shell; Fig. 4E).

###### Remarks.

The distinguishing characters of this species are the flattened shell and spire with a distinct suture. Shell surface nearly smooth with thin growth lines, varices present; following whorls regularly coiled. Last whorl angular, intermediately expanded; umbilicus very wide and showing all preceding whorls. Aperture semi-ovate with sinulus; peristome thickened, expanded and reflected; apertural dentition with only one parietal lamella (Fig. 4E).

*Discartemon sykesi* differs from *Discartemon discus* in its smaller shell, in the presence of a sinulus, the intermediately expanded last whorl, and in being restricted to the Malay Peninsula. This species can be distinguished from *Discartemon planus* in having a larger shell with flattened spire, the last whorl angular and intermediately expanded, a semi-ovate aperture, and in lacking palatal and columellar lamellae.

##### 4. Discartemon nummus

(Laidlaw, 1929)

http://species-id.net/wiki/Discartemon_nummus

**Figure 2.**
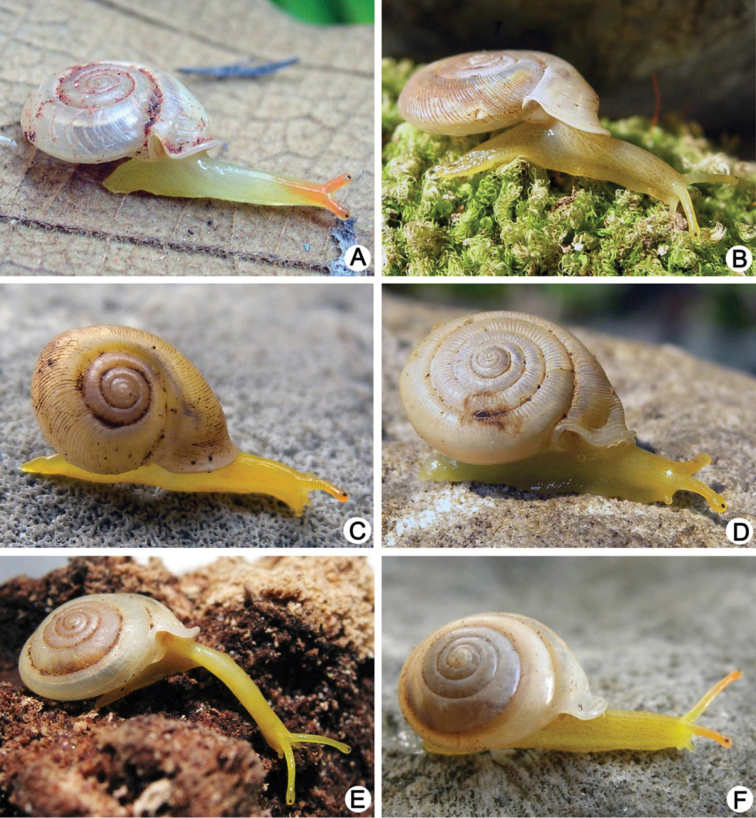
Living snails. **A** *Discartemon nummus* CUMZ 6208, from Patthalung (shell width about 6 mm) **B** *Discartemon discadentus* sp. n. paratype CUMZ 6209 (shell width about 12 mm) **C** *Discartemon leptoglyphus* CUMZ 6007, from Ipoh, Perak, Malaysia (shell width about 6 mm) **D** *Discartemon hypocrites* topotype CUMZ 6199 (shell width about 6 mm) **E, F** *Discartemon afthonodontia* sp. n. **E** paratype CUMZ 6210 (shell width about 9 mm), and **F** specimen CUMZ 6214, from Tam Khao Phlu, Chumphon (shell width about 8 mm).

Figs 2A , 4G , 11D, E , 17A–E , 22B , 23 , Table 1

Odontartemon (Discartemon) nummus Laidlaw, 1929: 259, 260, fig. 1. Type locality: Tale Sap, Singgora. Laidlaw 1933: 234.
Discartemon nummus – Benthem Jutting 1954: 87, 88. Benthem Jutting 1959: 168. Richardson 1988: 183.

###### Material examined.

Khao Ok Thalu, Phatthalung, Thailand (7°37'39.1"N, 100°5'19.1"E): CUMZ 3594 (24 shells; Fig. 4G) and 6208 (12 specimens in ethanol; Figs 2A, 11D, E, 17A–E, 22B).

###### Description.

**Shell.** Shell flattened, white and semi-transparent; whorls 5½, spire flattened with distinct suture. Shell surface glossy with thin transverse ridges near suture and varices present. Embryonic shell about 2½ whorls; following whorls regularly coiled. Last whorl angular with strong peripheral keel, regularly expanded; umbilicus very wide and showing all preceding whorls. Aperture triangular with sinulus; peristome continuous, thickened, expanded and reflected. Apertural dentition with a Y-shaped parietal lamella adjoining at sinulus (Fig. 4G).

**Radula.** Each row consists of 39–41 teeth with formula (19-20)-1-(19-20). Central tooth very small and triangular with pointed cusp. Lateral and marginal teeth undifferentiated, unicuspid and lanceolate. Latero-marginal teeth gradually reduce in size, with outermost teeth smaller and shorter than inner teeth (Fig. 22B).

**Genital organs.** Atrium (at) long. Proximal penis (p) long, slender; distal penis globularly enlarged. Penial sheath (ps) thin, extending about two-thirds of penis length; penial sheath retractor muscle very thin (psr), originating at genital orifice wall and inserting distally on penial sheath (Fig. 11D). Vas deferens (vd) passes through about one-fifth of penial sheath length before entering into penis distally (Fig. 11E). Penial retractor muscle (pr) thin and very long, inserting at penis and vas deferens junction.

Internal wall of atrium generally smooth (Fig. 17A); penial wall with scattered, short and transparent penial hooks, about 5 hooks/200 µm^2^ (Fig. 17B); hooks located on round-ovate penial papilla. Penial hooks of small size (<0.04 mm in length), slightly expanded at base, tip obtuse and directed towards genital orifice (Fig. 17C, D).

Vagina (v) short and stout, about half of penis length. Gametolytic duct (gd) a long tube extending as far as albumin gland; gametolytic sac (gs) ovate. Free oviduct (fo) long and thick; oviduct (ov) enlarged and folded; prostate gland inconspicuous and bound to oviduct. Talon (ta) small, very short and club shaped. Hermaphroditic duct (hd) bearing long seminal vesicle (sv) about one and half times longer than the length from talon to branching point of seminal vesicle (Fig. 11D).

Vaginal wall generally with smooth surface of longitudinal vaginal folds (Fig. 17E).

###### Remarks.

*Discartemon nummus* was described from Tale Sap (= Lake or Lagoon), Singgora (= Songkhla). In this study, living snails were found at an isolated limestone hill near the lake in Phatthalung, about 60 km north of the type locality.

Having the smallest shell size clearly discriminates *Discartemon nummus* from all congeners. It is similar to *Discartemon discus*, which has a larger shell and a peripheral keel, lacks a sinulus, and has a semi-ovate aperture with a straight parietal lamella. *Discartemon nummus* can be distinguished from *Discartemon khaosokensis* in having a flattened spire, the last whorl regularly expanded, a triangular aperture, and a Y-shaped parietal lamella.

##### 5. Discartemon khaosokensis

Panha & Burch, 1998

http://species-id.net/wiki/Discartemon_khaosokensis

Figs 4H , 23 , Table 1

Discartemon khaosokensis Panha & Burch, 1998: 25, 26, fig. 2. Type locality: Khao Sok National Park, Suratthani, Thailand.

###### Material examined.

Holotype CUMZ 6242 (Fig. 4H). Measurement: shell height 3.6 mm, shell width 11.4 mm, and with 5¾ whorls. Paratype CUMZ 6243 (4 shells).

###### Remarks.

This species is known only from the type locality. The shell is flattened and semi-transparent and has a concave spire with a distinct suture. Shell surface with transverse ridges that diminish below periphery, with varices present; whorls regularly coiled. Last whorl angular with a strong peripheral keel, rapidly expanded; umbilicus very wide, showing all preceding whorls. Aperture semi-ovate with narrow sinulus; peristome thin and expanded; apertural dentition of only one parietal lamella (Fig. 4H).

*Discartemon khaosokensis* differs from *Discartemon discus* in having a smaller shell, concave spire, a shell surface with tranverse ridges, a rapidly expanded last whorl with a strong peripheral keel, and a sinulus. *Discartemon khaosokensis* is also similar to *Discartemon sykesi*, but has a larger shell, a concave spire with tranverse ridges, and a rapidly expanded last whorl with a strong peripheral keel.

##### 6. Discartemon discadentus

Siriboon & Panha sp. n.

http://zoobank.org/E19CE74B-1858-4813-86E4-EACA08E703F0

http://species-id.net/wiki/Discartemon_discadentus

Figs 2B , 4I, J , 12A, B , 17F–I , 23 , Table 1

###### Type material.

Holotype CUMZ 6244 (Fig. 4I). Measurement: shell height 4.5 mm, shell width 12.1 mm, and with 6 whorls. Paratypes: CUMZ 6003 (2 shells), 6209 (1 specimen in ethanol; Figs 2B, 12A, B, 17F–I), 6258 (4 shells), NHMUK 20130672 (1 shell), and SMF (1 shell) from the type locality.

###### Type locality.

Wat Tam Yai, Thachana, Suratthani, Thailand (9°32'21.5"N, 99°11'29.4"E).

###### Diagnosis.

This new species can be distinguished from *Discartemon discus* and *Discartemon sykesi* by having transverse ridges that diminish below the periphery, and having an apertural dentition with five lamellae. In comparison, *Discartemon sykesi* has a smaller shell and *Discartemon discus* has a higher spire. The genitalia of *Discartemon discus* have a short penis, penial sheath and free oviduct, and long vagina while *Discartemon discadentus* sp. n. has a very long penis, penial sheath and free oviduct, and short vagina. *Discartemon discadentus* sp. n. differs from *Discartemon nummus* and *Discartemon khaosokensis* in having a larger shell with higher spire, in lacking a peripheral keel, and in usually having five apertural lamellae. The last whorl of *Discartemon khaosokensis* is rapidly expanded, while *Discartemon nummus* has a regularly expanded last whorl and Y-shaped parietal lamella. The genitalia of *Discartemon discadentus* sp. n. differ from those of *Discartemon nummus* in the long and slender penis, penial wall with reticulated folds, and long penial hooks located on conical penial papillae.

###### Description.

**Shell.** Shell flattened, white and translucent; whorls 6; spire only slightly elevated; suture distinct. Shell surface glossy with transverse ridges that diminish below periphery; varices present. Embryonic shell large, about 2½ whorls, with smooth surface; following whorls regularly coiled. Last whorl angular, intermediately expanded; umbilicus very wide and showing all preceding whorls. Aperture semi-ovate; peristome discontinuous, thickened and expanded. Apertural dentition usually with one strong parietal, one palatal, one small basal and one strong columellar lamella. A small supracolumellar lamella is sometimes present (Fig. 4I).

**Genital organs.** Atrium (at) short; penis (p) very long and slender. Penial sheath (ps) thin, extending about five-sixths of penis length; penial sheath retractor muscle very thin (psr), originating at genital orifice wall and inserting distally on penial sheath (Fig. 12A). Vas deferens (vd) passes through a very short part of penial sheath before entering into penis distally (Fig. 12B). Penial retractor muscle (pr) thin and very long, inserting at penis and vas deferens junction.

Internal wall of atrium generally corrugated (Fig. 17F). Penial wall with scattered and transparent penial hooks, about 6 hooks/200 µm^2^ (Fig. 17G); hooks located on conical penial papillae (pp) separated by low reticulated folds. Penial hooks small (<0.03 mm in length), expanded at base, tips pointed and curved towards genital orifice (Fig. 17H).

Vagina (v) short, about one seventh of penis length. Gametolytic duct (gd) a long tube extending as far as albumin gland; gametolytic sac (gs) ovate. Proximal free oviduct (fo) convoluted and distally long and thick; oviduct (ov) enlarged and folded. Prostate gland inconspicuous and bound to oviduct. Talon (ta) small, short and club shaped. Hermaphroditic duct (hd) bearing long seminal vesicle (sv) about four times longer than the length from talon to branching point of seminal vesicle (Fig. 12A).

Vaginal wall generally with smooth surface of reticulated vaginal folds (Fig. 17I).

###### Etymology.

The specific epithet “*discadentus*” is derived from the Latin “*discus*” meaning “disc” and “*dentatus*” meaning “teeth”.

###### Distribution.

This species seems to be restricted to limestone areas in Suratthani Province, Thailand. Tam Khuha, Kanchanadit District, is an isolated limestone hill about 40 km southeast of the type locality and Wat Tam Wararam, Phanom District, is in the limestone mountains near Ratchaprapa Dam, about 70 km southwest of the type locality.

###### Remarks.

This species shows variation in shell size and the presence of the infrapalatal, upper palatal and supracolumellar lamellae. Some specimens from Phanom, Suratthani (CUMZ 3571, 3582) possess an upper palatal and supracolumellar lamella, and an infrapalatal lamella is present in one paratype shell (CUMZ 6003). Populations from Tam Khuha, Suratthani (CUMZ 6004) exhibit a relatively smaller shell size (width about 11 mm). This new species is apparently rare and only extensive searching revealed living animals.

##### 7. Discartemon discamaximus

Siriboon & Panha sp. n.

http://zoobank.org/EE36EC6D-DDE1-420E-A325-CDF9C11EA5BB

http://species-id.net/wiki/Discartemon_discamaximus

Figs 5A, B , 23 , Table 1

###### Type material.

Holotype CUMZ 6245 (Fig. 5A). Measurement: shell height 4.7 mm, shell width 14.6 mm, and with 7 whorls. Paratypes: CUMZ 6005 (2 shells) and NHMUK 20130673 (2 shells) from the type locality.

###### Other material examined.

Tam Kobe, Phangnga: CUMZ 3669, 6197.

###### Type locality.

Tam Namphud, Phangnga, Thailand, 8°27'46.8"N, 98°32'30.5"E.

###### Diagnosis.

The characters distinguishing *Discartemon discamaximus* sp. n. from *Discartemon sykesi* and *Discartemon khaosokensis* are the larger shell with flattened to concave spire, the transverse ridges present near the suture, and the lack of a sinulus. *Discartemon discamaximus* sp. n. has similar shell morphology to *Discartemon discus* and *Discartemon discadentus* sp. n., but is distinguished by having the transverse ridges present only near the suture and the last whorl rapidly expanded. *Discartemon discadentus* sp. n. also has five apertural lamellae.

###### Description.

**Shell.** Shell flattened, white and translucent; whorls 7, spire flattened to concave, with distinct suture. Shell surface glossy with transverse ridges near suture and varices present. Embryonic shell large, about 2½ whorls, with smooth surface; following whorls regularly coiled. Last whorl angular, rapidly expanded; umbilicus very wide and showing all preceding whorls. Aperture semi-ovate; peristome discontinuous, expanded and reflected; apertural dentition with one parietal lamella (Fig. 5A).

###### Etymology.

The specific epithet “*discamaximus*” is derived from the Latin “*discus*” meaning “disc” and “*maximus*” meaning “large or broad”.

###### Distribution.

This new species is known from limestone karst near Phanganga Bay reaching about 100–400 meters amsl, surrounded by the Phuket mountain range.

###### Remarks.

To date no living specimens have been found.

##### 8. Discartemon circulus

Siriboon & Panha sp. n.

http://zoobank.org/C9CFC89A-F272-45C8-AF3C-1612828174D4

http://species-id.net/wiki/Discartemon_circulus

Figs 5C, D , 23 , Table 1

###### Type material.

Holotype CUMZ 6246 (Fig. 5C). Measurement: shell height 3.9 mm, shell width 7.7 mm, and with 6 whorls. Paratypes: CUMZ 3665 (9 shells), 6262 (8 shells), NHMUK 20130674 (2 shells), and SMF (2 shells) from the type locality.

###### Type locality.

Tam Phannara, Nakhon Si Thammarat, Thailand, 8°25'18.8"N, 99°22'46.8"E.

###### Diagnosis.

*Discartemon circulus* sp. n. differs from *Discartemon discus* and *Discartemon sykesi* in its narrower umbilicus, sub-quadrangular aperture, and apertural dentition with five lamellae. In addition, *Discartemon discus* has a larger shell, while *Discartemon sykesi* has an intermediately expanded last whorl and a sinulus. Compared with *Discartemon khaosokensis*, *Discartemon circulus* sp. n. has a smaller shell, a flattened spire with weak transverse ridges, an angular last whorl, a sinulus, and five apertural lamellae. *Discartemon circulus* sp. n. differs from *Discartemon discadentus* sp. n. and *Discartemon discamaximus* sp. n. in having a smaller shell with weak transverse ridges and five apertural lamellae. Compared with *Discartemon expandus* sp. n., *Discartemon circulus* sp. n. has weaker transverse ridges, a regularly expanded peristome, and five apertural lamellae.

###### Description.

**Shell.** Shell flattened, white and translucent; whorls 5½–6, spire flattened, with a distinct suture. Shell surface glossy with weak transverse ridges and varices present. Embryonic shell large, about 2½ whorls, with a smooth surface; following whorls regularly coiled. Last whorl angular, regularly expanded; umbilicus very wide, deep and showing all preceding whorls. Aperture sub-quadrangular; peristome discontinuous, thin and expanded. Apertural dentition with one parietal, one palatal, one small basal, one small columellar and one small supracolumellar lamella (Fig. 5C).

###### Etymology.

The specific epithet is from the Latin “*circulus*” meaning “circle”. It refers to the appearance of this new species when seen from the apex.

###### Distribution.

This species is known only from the type locality, an isolated limestone hill which reaches about 200 meters amsl, about 20 km southwest of Tai Rom Yen National Park.

###### Remarks.

Apparently rare and extensive searching revealed no living examples.

##### 9. Discartemon deprima

Siriboon & Panha sp. n.

http://zoobank.org/B7EAE186-FD2D-40CB-BEFE-72E1CD7A5684

http://species-id.net/wiki/Discartemon_deprima

Figs 5E, F , 23 , Table 1

###### Type material.

Holotype CUMZ 6247 (Fig. 5E). Measurement: shell height 2.5 mm, shell width 8.2 mm, and with 5 whorls. Paratypes: CUMZ 3573 (2 shells), NHMUK 2013675 (1 shell), and SMF (1 shell) from the type locality. Paratype: CUMZ 6259 from Ban Tam Thong, Prathiew, Chumphon.

###### Other material examined.

Khao Pu-Khao Ya National Park, Sri Banphot, Phatthalung: CUMZ 3670.

###### Type locality.

Khao Hup Ta Hae, Prathiew, Chumphon, Thailand, 10°48'44.9"N, 99°25'9.0"E.

###### Diagnosis.

This species closely resembles *Discartemon sykesi*, but is distinct in having a concave spire and strong peripheral keel on the last whorl. Compared with *Discartemon khaosokensis*, *Discartemon deprima* sp. n. has a smaller shell with weaker transverse ridges, and the last whorl intermediately expanded. *Discartemon deprima* sp. n. differs from *Discartemon nummus* by having a larger shell, a concave spire, and one straight parietal lamella. It differs from *Discartemon circulus* sp. n. and *Discartemon expandus* sp. n. in having a concave spire, the last whorl intermediately expanded with a strong peripheral keel, and in having only one parietal lamella and a sinulus. In addition, *Discartemon expandus* sp. n. has transverse ridges that diminish below the periphery, and has a thin and widely expanded peristome.

###### Description.

**Shell.** Shell flattened, white and semi-transparent; whorls 5, spire concave with a distinct suture. Shell surface glossy with weak transverse ridges that diminish below periphery and appear again near peristome; varices present. Embryonic shell large, about 2½ whorls, with a smooth surface; following whorls regularly coiled. Last whorl angular with strong peripheral keel, intermediately expanded. Umbilicus very wide and showing all preceding whorls. Aperture semi-ovate with sinulus; peristome discontinuous, thin, expanded and reflected. Apertural dentition with only one parietal lamella (Fig. 5E).

###### Etymology.

The specific epithet “*deprima*” is derived from the Latin “*deprimo*” meaning “depress”. It refers to the depressed spire of this new species.

###### Distribution.

This species is known from the east coast of Chumphon, on an isolated limestone hill reaching about 200 meters amsl, and from a more southerly locality in Patthalung, a limestone hill complex reaching about 200–400 meters amsl.

###### Remarks.

There is some variation in this species in the discontinuous peristome and the presence of a sinulus. The samples from Patthalung (CUMZ 3670, 2 shells) have a continuous peristome and lack a sinulus. Currently, no living examples have been found.

##### 10. Discartemon expandus

Siriboon & Panha sp. n.

http://zoobank.org/E1B561EF-83BF-4D71-A5BD-4F4C9A839F84

http://species-id.net/wiki/Discartemon_expandus

Figs 5G, H , 23 , Table 1

###### Type material.

Holotype CUMZ 6248 (Fig. 5G). Measurement: shell height 3.8 mm, shell width 8.3 mm, and with 5½ whorls. Paratypes: CUMZ 3664 (10 shells), NHMUK 20130676 (2 shells), and SMF (2 shells) from the type locality.

###### Type locality.

Klong Hoy, Suratthani, Thailand, 8°57'18.1"N, 98°48'30.7"E.

###### Diagnosis.

*Discartemon expandus* sp. n. differs from *Discartemon discus* and *Discartemon sykesi* in its smaller shell with transverse ridges, intermediately expanded last whorl, and widely expanded peristome. In addition, a sinulus is absent in *Discartemon discus*. *Discartemon expandus* sp. n. can be distinguished from *Discartemon khaosokensis* by having a flattened spire, an angular and intermediately expanded last whorl, and a widely expanded peristome. Compared with *Discartemon discadentus* sp. n. and *Discartemon discamaximus* sp. n., *Discartemon expandus* sp. n. has a smaller shell with strong transverse ridges, a sinulus and a widely expanded peristome. Moreover, *Discartemon discadentus* sp. n. has five apertural lamellae, and *Discartemon discamaximus* sp. n. has a rapidly expanded last whorl.

###### Description.

**Shell.** Shell flattened, white and semi-transparent; whorls 5½–6, spire flattened with a distinct suture. Shell surface glossy with transverse ridges that diminish below periphery; varices present. Embryonic shell large, about 2–2½ whorls, with a smooth surface; following whorls regularly coiled. Last whorl angular, intermediately expanded; umbilicus very wide, deep and showing all preceding whorls. Aperture semi-ovate with narrow sinulus; peristome discontinuous, thin and widely expanded. Apertural dentition of only one parietal lamella (Fig. 5G).

###### Etymology.

The specific epithet “*expandus*” is derived from the Latin “*expandi*” meaning “expand”. It refers to the expanded peristome of this species.

###### Distribution.

The species is known only from the type locality and extensive searching revealed no living examples.

###### Remarks.

Some variation has been observed in the spire, which is slightly convex rather than flattened in some specimens, and in the distinctness of the suture.

#### Group II: *Discartemon plussensis*-group: Species with depressed-heliciform shell.

##### 11. Discartemon plussensis

(Morgan, 1885)

http://species-id.net/wiki/Discartemon_plussensis

Figs 6A, B , 23 , Table 2

Streptaxis plussensis Morgan, 1885a [Jan.]: 68. Type locality: Mont Tchéhèl, dans la Vallée de la rivière Pluss. Morgan 1885b [Aug.]: 371, 372, pl. 5, fig. 1. Tryon 1885: 251. Möllendorff 1887: 299, 300. Tenison-Woods 1888: 1009. Möllendorff 1891: 330, 331. Gude 1903: 226.
Odontartemon (Discartemon) plussensis – Kobelt 1906: 99, pl. 54, figs 12–14. Kobelt 1910: 150.
Discartemon plussensis – Benthem Jutting 1954: 79, fig. 2. Benthem Jutting 1959: 168. Richardson 1988: 184. Maassen 2001: 87, 88.

###### Material examined.

Perak, Malaysia NHMUK 1939.4.13.22 (1 shell; Fig. 6A). Sungei Siput, Perak, Malaysia: NMW 1955.158.25253 (13 shells). RMNH Kaumans Reg. 598 (3 shells). Hot Springs, Tanjung Rambutan, Perak, Mlaysia: RMNH Drijver Coll. (1 shell). Yan Tao San, Perak, Malaysia: CUMZ 6008. Ipoh, Perak, Malaysia (4°36'34.6"N, 101°6'49.9"E): CUMZ 6009 (Fig. 6B; 3 shells).

**Figure 3.**
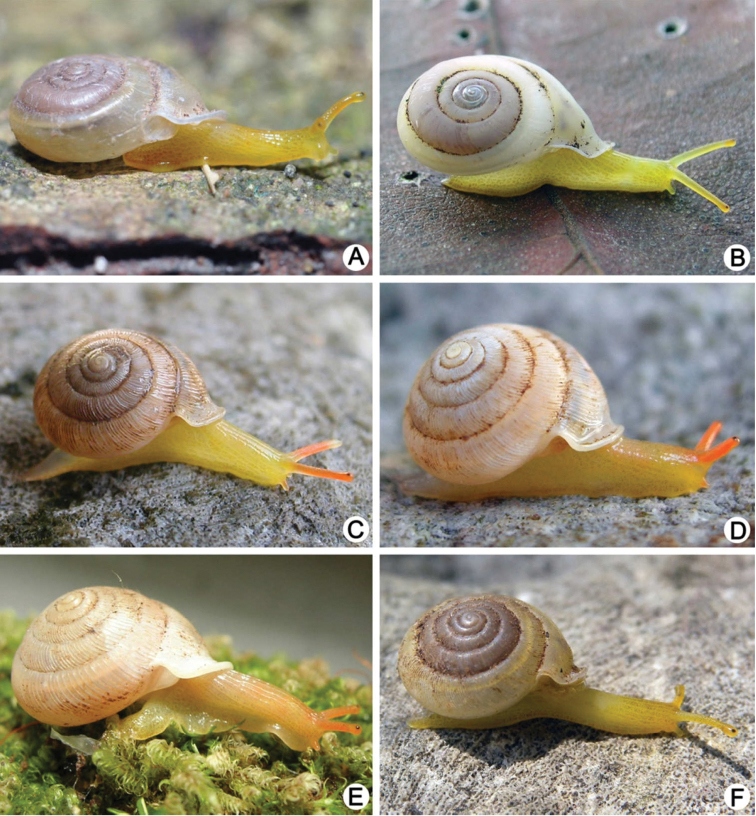
Living snails. **A** *Discartemon epipedis* sp. n. paratype CUMZ 6215 (shell width about 9 mm) **B** *Discartemon flavacandida* sp. n. paratype CUMZ 6216 (shell width about 12 mm)**C** *Discartemon roebeleni* topotype CUMZ 6217 (shell width about 9 mm) **D** *Discartemon kotanensis* sp. n. paratype CUMZ 6230 (shell width about 9 mm) **E** *Discartemon megalostraka* sp. n. CUMZ 6233, from Phangnga (shell width about 12 mm), and **F** *Discartemon triancus* sp. n. paratype CUMZ 6236 (shell width about 7 mm).

**Figure 4.**
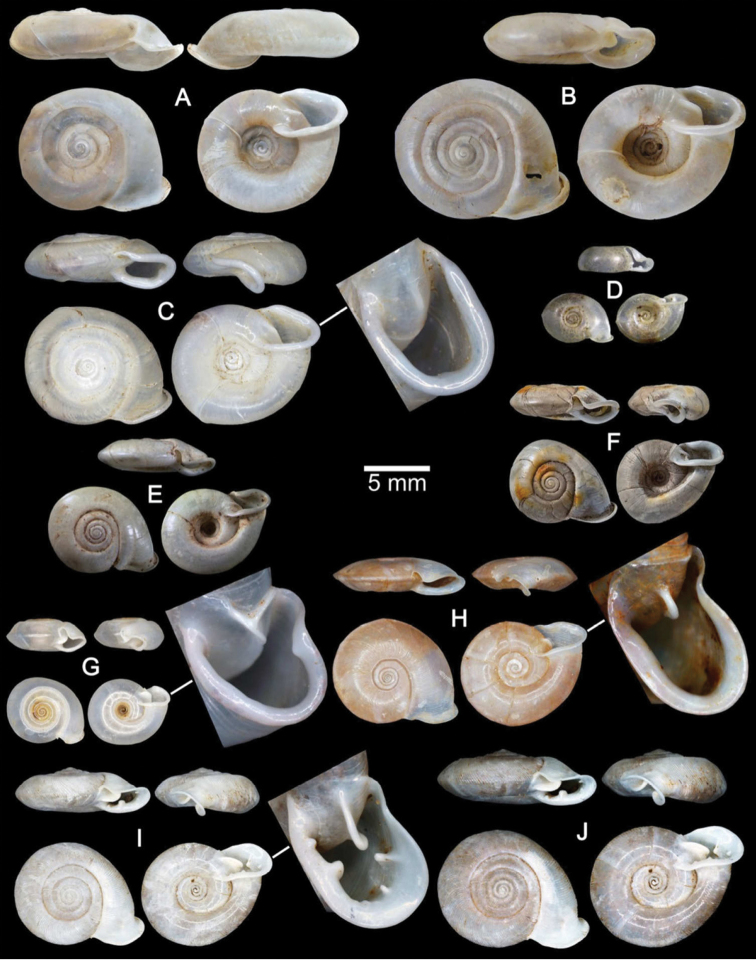
Shells of Group I: *Discartemon discus*-group. **A–C** *Discartemon discus* **A** holotype NHMUK 20130684 **B** lectotype SMF 108534 of “*Streptaxis paradiscus* Möllendorff, 1900”, and **C** specimen CUMZ 6001, from Vietnam with apertural dentition **D** *Discartemon planus* specimen NMW 1955.158.25252, from Sulawesi, Indonesia **E, F** *Discartemon sykesi* **E** paratype NMW 1955.158.25257, and **F** paratype NHMUK 1937.7.9.11 **G** *Discartemon nummus* CUMZ 3594, from Patthalung with apertural dentition **H** *Discartemon khaosokensis* holotype CUMZ 6242 with apertural dentition **I, J** *Discartemon discadentus* sp. n. **I** holotype CUMZ 6244 with apertural dentition, and **J** paratype CUMZ 6209.

**Figure 5.**
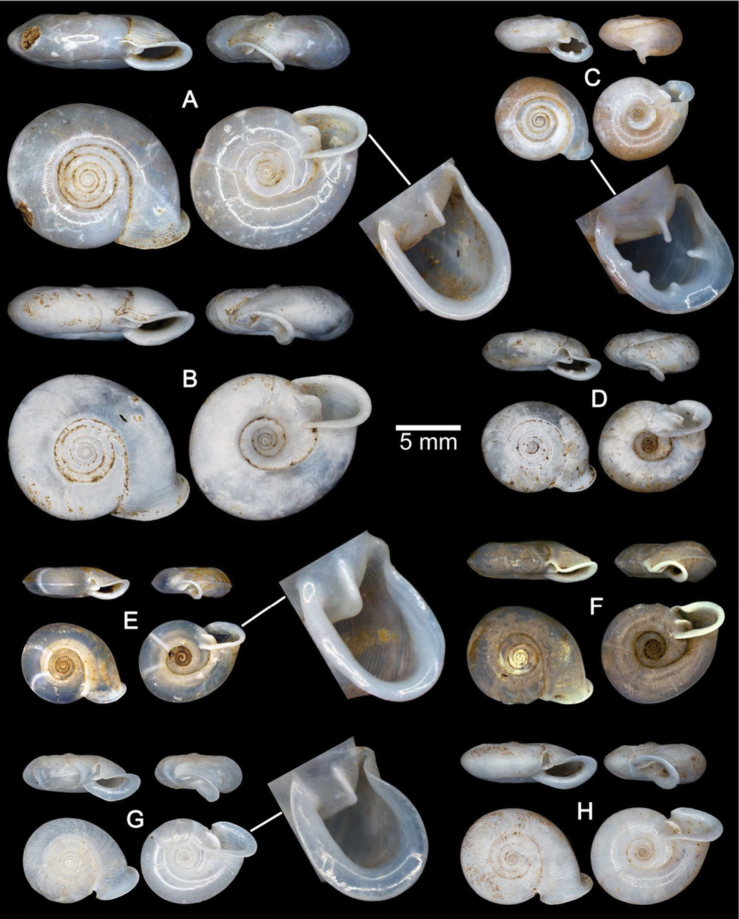
Shells of Group I: *Discartemon discus*-group. **A, B** *Discartemon discamaximus* sp. n. **A** holotype CUMZ 6245 with apertural dentition, and **B** paratype CUMZ 6005 **C, D** *Discartemon circulus* sp. n. **C** holotype CUMZ 6246 with apertural dentition, and **D** paratype CUMZ 3665 **E, F** *Discartemon deprima* sp. n. **E** holotype CUMZ 6247 with apertural dentition, and **F** paratype CUMZ 3573 **G, H** *Discartemon expandus* sp. n. **G** holotype CUMZ 6248 with apertural dentition, and **H** paratype CUMZ 3664.

**Figure 6.**
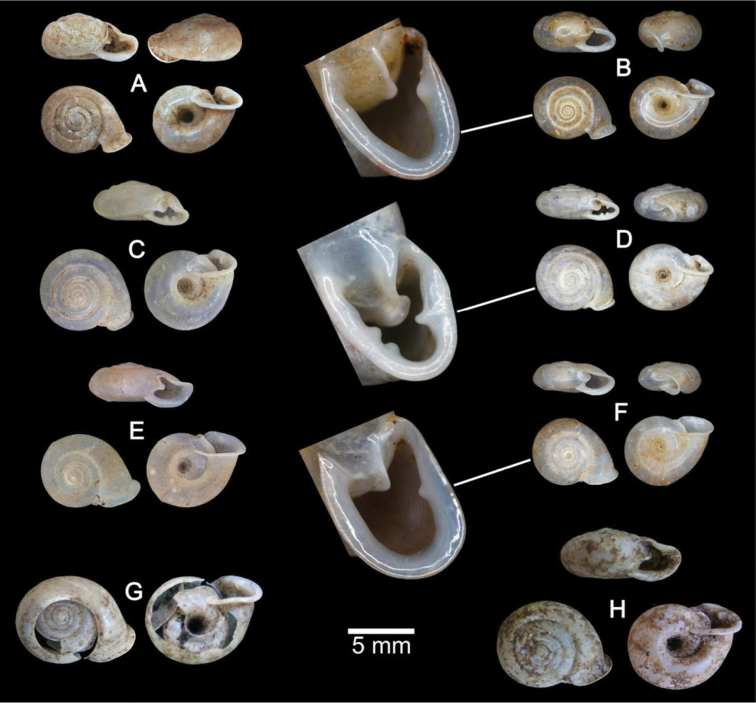
Shells of Group II: *Discartemon plussensis*-group. **A, B** *Discartemon plussensis* **A** specimen NHMUK 1939.4.13.22, from Perak, Malaysia, and **B** topotype CUMZ 6009 with apertural dentition **C, D** *Discartemon hypocrites* **C** holotype ZMA 3.34.017, and **D** topotype CUMZ 6011 with apertural dentition **E, F** *Discartemon leptoglyphus* **E** holotype ZMA 3.54.019, and **F** specimen CUMZ 6007, from Ipoh, Perak, Malaysia with apertural dentition **G, H** *Discartemon platymorphus* **G** holotype ZMA 3.54.022, and **H** paratype ZMA 3.54.023.

**Table 2.** Shell measurements of *Discartemon* spp (*Discartemon plussensis*-group). Specimen collections and catalogue numbers indicated in parentheses.

Species and locality and CUMZ nos	No. of specimens	Ranges, mean ± S.D. in mm of:			Number of whorls
		Shell height	Shell width	H/W ratio	
*Discartemon plussensis* (Morgan, 1885)					
Yan Tao San, Perak, Malaysia: (6008)	24	3.0–3.8 3.4±0.22	5.8–7.5 6.6±0.45	0.4–0.6 0.5±0.03	5½–6
Ipoh, Perak, Malaysia: (6009)	3	2.9–3.3 3.0±0.23	5.6–6.2 6.0±0.36	0.5–0.5 0.5±0.04	5½–6
*Discartemon hypocrites* Benthem Jutting, 1954					
Gaplu Bukit, Perlis, Malaysia: (6198)	4	2.8–3.0 2.9±0.08	6.5–7.3 6.9±0.41	0.4–0.4 0.4±0.02	5–5½
*Discartemon leptoglyphus* Benthem Jutting, 1954					
Lost World, Perak, Malaysia: (6007, 6260)	15	2.7–3.1 2.9±0.13	6.8–8.0 7.2±0.30	0.4–0.4 0.4±0.02	5–5½
Ampang Baru, Ipoh, Perak, Malaysia: (6010)	3	2.9–3.2 3.1±0.13	6.6–7.5 7.0±0.41	0.4–0.5 0.4±0.02	5–5½
*Discartemon afthonodontia* sp. n.					
Tam Phitsadan, Chumphon: (4206, 6019, 6249)	29	3.9–5.4 4.7±0.35	8.3–9.6 9.0±0.29	0.4–0.6 0.5±0.04	6
Tam Khao Phlu, Chumphon: (3581, 3666)	31	3.4–4.4 3.9±0.26	7.0–8.4 7.9±0.31	0.4–0.6 0.5±0.03	6
Khao Maeo, Chumphon: (3589)	13	3.6–4.3 3.9±0.20	7.3–8.1 7.8±0.23	0.4–0.5 0.5±0.02	6
Wat Tam Phru-Takien, Chumphon: (6016)	6	4.2–5.0 4.6±0.25	6.7–7.6 7.3±0.26	0.5–0.6 0.6±0.03	6
Wat Uthai Tam, Chumphon: (6261)	28	4.0–5.3 4.8±0.32	7.8–9.1 8.3±0.32	0.5–0.6 0.6±0.03	6
Bang Saphan Noi, Prachuap Khirikhan: (3588)	7	4.6–6.1 5.2±0.33	6.8–8.5 7.6±0.50	0.5–0.7 0.6±0.05	6
Wat Tam Khao Marong, Prachuap Khirikhan: (6014)	17	4.2–5.0 4.6±0.25	6.7–7.6 7.3±0.26	0.6–0.7 0.6±0.02	6
*Discartemon epipedis* sp. n.					
Gua Matu Madu, Kelantan, Malaysia: (6020, 6250)	20	4.1–5.2 4.5±0.25	8.2–9.1 8.6±0.24	0.5–0.6 0.5±0.04	6
*Discartemon flavacandida* sp. n.					
Tam Phra Khayang, Ranong: (3574, 3576, 3675, 3676, 4214, 6006, 6251)	130	4.6–6.0 5.2±0.36	10.1–12.9 11.2±0.51	0.4–0.5 0.5±0.03	6–6½

###### Remarks.

The original descriptions included informative figures (Morgan 1885a, b), and subsequently Benthem Jutting (1954: fig. 2) published excellent figures of topotype specimens. These allow unambiguous recognition of this species. Shell depressed-heliciform with a flattened spire. Shell surface with transverse ridges that diminish below periphery and varices present; following whorls regularly coiled. Last whorl rounded and regularly expanded; umbilicus widely open and deep. Aperture triangular with sinulus, and apertural dentition with one parietal and one palatal lamella (Fig. 6B).

Compared with *Discartemon leptoglyphus* and *Discartemon plussensis*, it differs in its smaller shell, with transverse ridges that diminish below periphery, last whorl rounded and more inflated, and sinulus present.

##### 12. Discartemon hypocrites

Benthem Jutting, 1954

http://species-id.net/wiki/Discartemon_hypocrites

Figs 2D , 6C, D , 12C, D , 18A–E , 22C , 23 , Table 2

Discartemon hypocrites Benthem Jutting, 1954: 92–94, fig. 8. Type locality: Bukit Chuping, Perlis, Malaysia. Benthem Jutting 1959: 168. Richardson 1988: 183. Maassen 2001: 87.

###### Material examined.

Holotype ZMA 3.34.017 (Fig. 6C). Paratypes: ZMA 3.54.018 (5 shells). Bukit Chuping, Perlis, Malaysia (6°29'36.2"N, 100°15'53.2"E): CUMZ 6011 (2 shells; Fig. 6D). Guplu Bukit, Perlis, Malaysia: CUMZ 6198. Kaki Bukit, Perlis, Malaysia: CUMZ 6199 (1 specimen in ethanol; Figs 2D, 12C, D, 18A–E, 22C).

###### Description.

**Shell.** Shell depressed-heliciform, white and semi-transparent; whorls 5–5½, spire only slightly convex with distinct suture. Shell surface glossy with thin transverse ridges that diminish below periphery; varices present. Whorls regularly coiled; last whorl rounded, regularly expanded; umbilicus very wide, deep and showing all preceding whorls. Aperture triangular; peristome discontinuous, thickened, expanded and reflected. Apertural dentition with one sinuous parietal, one palatal, one columellar and one supracolumellar lamella (Fig. 6C).

**Radula.** Each row consists of 43 teeth with formula (21)-1-(21). Central tooth very small and triangular with a pointed cusp. Lateral and marginal teeth undifferentiated, unicuspid and lanceolate. Latero-marginal teeth gradually reduce in size, with outermost teeth much smaller and shorter than inner teeth (Fig. 22C).

**Genital organs.** Atrium (at) long and thick. Proximal penis (p) with short and stout penial appendix (pa) about two-thirds of penis length; distal penis slender (Fig. 12C). Penial sheath retractor muscle (psr) very thin, originating at genital orifice wall and inserting distally on penial sheath (Fig. 12C). Vas deferens (vd) passes through about a quarter of penial sheath length before entering into penis distally (Fig. 12D). Penial retractor muscle (pr) thin and very long, inserting at penis and vas deferens junction.

Internal wall of atrium generally smooth with numerous atrial pores (Fig. 18A). Penial wall with dense and brownish penial hooks, about 4 hooks/200 µm^2^ (Fig. 18B). Hooks located on laterally-flattened penial papillae (pp), which are separated by thin reticulated folds. Penial hooks very small (< 0.01 mm in length), expanded at base, pointed at tip and curved towards genital orifice (Fig. 18C, D).

Vagina (v) short. Gametolytic duct (gd) enlarged and stout at base, and suddenly tapering to small and long tube extending as far as albumin gland; gametolytic sac (gs) ovate. Free oviduct (fo) proximally large with equivalent diameter to vagina, tapering to smaller tube distally. Oviduct (ov) enlarged and folded; prostate gland inconspicuous and bound to oviduct. Talon (ta) small, short and club shaped. Hermaphroditic duct (hd) bearing long and thick seminal vesicle (sv) about one and half times longer than the length from talon to branching point of seminal vesicle (Fig. 12C).

Vaginal wall with reticulated vaginal folds (Fig. 18E).

###### Distribution.

This species is known from several limestone hills in Perlis, Malaysia.

###### Remarks.

*Discartemon hypocrites* can be distinguished from *Discartemon plussensis*, *Discartemon leptoglyphus* and *Discartemon platymorphus* by the apertural dentition with one sinuous parietal, one columellar, and one supracolumellar lamella. The latter three species exhibit one straight parietal and one palatal lamella. In addition, *Discartemon plussensis* has a lower spire, an inflated last whorl and a sinulus; *Discartemon leptoglyphus* has transverse ridges over the entire shell; and *Discartemon platymorphus* has a larger shell and lower spire. *Discartemon hypocrites* also differs from *Discartemon leptoglyphus* in having a slender penis with short and stout penial appendix, the vas deferens passing through about a quarter of penial sheath length, the pointed penial hooks located on laterally-flattened penial papillae, and the vagina having reticulated folds.

##### 13. Discartemon leptoglyphus

Benthem Jutting, 1954

http://species-id.net/wiki/Discartemon_leptoglyphus

Figs 2C , 6E, F , 13A, B , 18F–L , 23 , Table 2

Discartemon leptoglyphus Benthem Jutting, 1954: 90–92, fig. 7. Type locality: Gunong Rapat, near Ipoh, Perak, Malaysia. Benthem Jutting 1959: 168. Maassen 2001: 87.

###### Material examined.

Holotype ZMA 3.54.019 (Fig. 6E). Paratypes NHMUK 1954.4.3.3 (1 shell), ZMA 3.54.020 (1 shell), ZMA 3.54.021 (5 shells). Ampang Baru, Ipoh, Perak, Malaysia, 6°29'36.2"N, 100°15'53.2"E, CUMZ 6010 (3 shells; Fig. 6F). Lost World, Tanjung Rambutan, Ipoh, Perak, Malaysia: CUMZ 6007 (9 specimens in ethanol; Figs 2C, 13A, B, 18F–L), 6260 (4 shells).

###### Description.

**Shell.** Shell depressed-heliciform, white and semi-transparent; whorls 5–5½, spire only slightly convex with distinct suture. Shell surface glossy with transverse ridges and varices present. Whorls regularly coiled; last whorl angular, regularly expanded, ultimate part expanded; umbilicus very wide, deep and showing all preceding whorls. Aperture triangular, sometimes semi-ovate; peristome discontinuous, expanded and little reflected. Apertural dentition of one parietal and one small palatal lamella (Fig. 6E).

**Genital organs.** Atrium (at) short. Penis (p) long, swollen at middle and with a long and slender penial appendix (pa) about half of penis length. Penial sheath (ps) thin, extending about one-third of penis length; penial sheath retractor muscle (psr) very thin, originating at genital orifice wall and inserting distally on penial sheath (Fig. 13A). Vas deferens (vd) passes through entire length of penial sheath before entering into penis distally (Fig. 13B). Penial retractor muscle (pr) thin and very long, inserting at penis and vas deferens junction.

Internal wall of atrium with smooth surface (Fig. 18F). Penial wall with translucent penial hooks densely scattered, about 18 hooks/200 µm^2^ (Fig. 18G). Hooks located on ovate penial papillae (pp). Penial hooks small (< 0.04 mm in length), tips obtuse and curved towards genital orifice (Fig. 18H–K).

Vagina (v) short, about one-third of penis length. Proximal gametolytic duct (gd) enlarged, stout; distally a long tube extending as far as albumin gland; gametolytic sac (gs) ovate. Proximal free oviduct (fo) enlarged then tapering to smaller tube distally. Oviduct (ov) enlarged and folded; prostate gland inconspicuous and bound to oviduct. Talon (ta) small and club shaped. Hermaphroditic duct (hd) bearing long and thick seminal vesicle (sv) about one and half times longer than the length from talon to branching point of seminal vesicle (Fig. 13A).

Vaginal wall generally smooth (Fig. 18L).

###### Distribution.

This species is known from the limestone mountains around the type locality in Perak, Malaysia.

###### Remarks.

Compared with *Discartemon platymorphus*, this species differs in having a smaller shell, with transverse ridges appearing on the entire shell and a more inflated last whorl. *Discartemon leptoglyphus* can be distinguished from *Discartemon stenostomus* by having a depressed-heliciform shell with lower spire, transverse ridges on the entire shell, the last whorl angular, and apertural dentition of one straight parietal lamella. In addition, the penial appendix in *Discartemon leptoglyphus* is relatively much longer than that shown for *Discartemon stenostomus* (see Berry 1965).

##### 14. Discartemon platymorphus

Benthem Jutting, 1954

http://species-id.net/wiki/Discartemon_platymorphus

Figs 6G, H , 23

Discartemon platymorphus Benthem Jutting, 1954: 88-90, fig. 6. Type locality: Gua Nenek, Kelantan, Malaysia. Benthem Jutting 1959: 168. Berry 1965: 28, 29. Richardson 1988: 184. Maassen 2001: 87. Marwoto 2008: 192.

###### Material examined.

Holotype ZMA 3.54.022 (fragmented) (Fig. 6G). Paratype ZMA 3.54.023 (2 shells).

###### Remarks.

The shell is depressed-heliciform with the spire only slightly convex and with a distinct suture. The shell surface has transverse ridges that diminish below the periphery, and varices are present. The following whorls are regularly coiled. Last whorl rounded, regularly expanded; umbilicus widely open and deep. Aperture triangular; peristome discontinuous, thickened, expanded and little reflected; apertural dentition of one parietal and one palatal lamella (Fig. 6H).

*Discartemon platymorphus* is closely similar to *Discartemon plussensis*, but that species has a larger shell with a higher spire and lacks a sinulus. *Discartemon platymorphus* differs from *Discartemon epipedis* sp. n. by having a lower spire, transverse ridges that diminish below the periphery, a shouldered last whorl, and apertural dentition with four lamellae. Compared with *Discartemon stenostomus*, *Discartemon platymorphus* has a lower spire with fine transverse ridges that disappear below the periphery, and a straight parietal lamella and one palatal lamella.

##### 15. Discartemon afthonodontia

Siriboon & Panha sp. n.

http://zoobank.org/423A1CDB-BEE3-4E34-B120-65F89617A73F

http://species-id.net/wiki/Discartemon_afthonodontia

**Figure 7.**
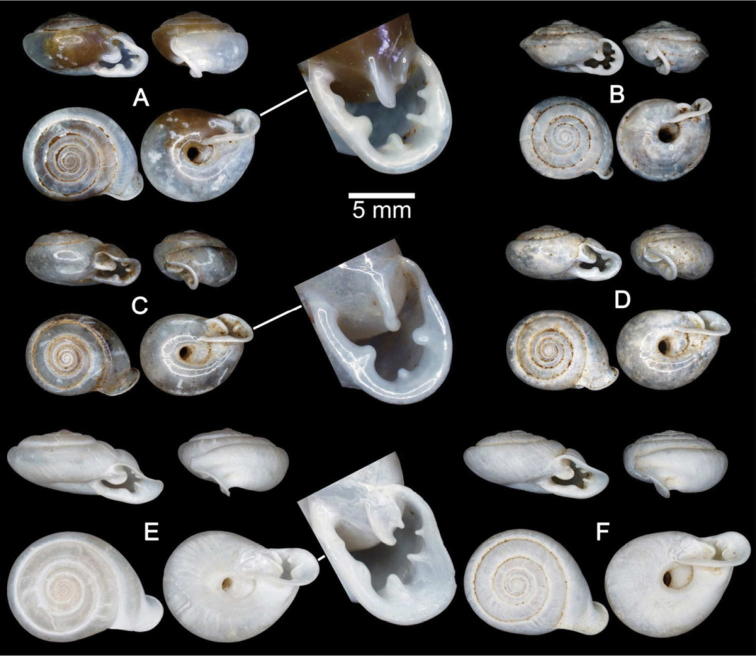
Shells of Group II: *Discartemon plussensis*-group. **A, B** *Discartemon afthonodontia* sp. n. **A** holotype CUMZ 6249 with apertural dentition, and **B** specimen CUMZ 3581, from Tam Khao Phlu, Chumphon **C, D** *Discartemon epipedis* sp. n. **C** holotype CUMZ 6250 with apertural dentition, and **D** paratype CUMZ 6020 **E, F** *Discartemon flavacandida* sp. n. **E** holotype CUMZ 6251 with apertural dentition, and **F** paratype CUMZ 3574.

Figs 2E, F , 7A, B , 13C–F , 19A–E , 22D , 23 , Table 2

###### Type material.

Holotype CUMZ 6249 (Fig. 7A). Measurement: shell height 4.8 mm, shell width 9.3 mm, and with 6½ whorls. Paratypes: CUMZ 4206 (1 shell), 6018 (4 shells), 6019 (23 shells), 6210 (7 specimens in ethanol; Figs 2E, 13C–F, 19A–E, 22D), NHMUK 20130677 (2 shells), and SMF (2 shells) from the type locality.

###### Other material examined.

Wat Khao Pho, Bang Saphan, Prachuap Khirikhan: CUMZ 6012, 6013. Wat Tam Khao Marong, Bang Saphan, Prachuap Khirikhan: CUMZ 4219, 6014, 6211 (5 specimens in ethanol). Bang Saphan Noi, Prachuap Khirikhan: CUMZ 3588. Tam Khao Phlu, Prathiew, Chumphon: CUMZ 3581, 3666, 6214 (3 specimens in ethanol; Figs 2F, 7B). Khao Maeo, Prathiew, Chumphon: CUMZ 3589. Nam Tok Kapo, Tha Sae, Chumphon: CUMZ 3593. Wat Tam Phru-Takien, Tha Sae, Chumphon: CUMZ 6016. Wat Uthai Tam, Chumphon: CUMZ 6212 (6 specimens in ethanol), 6261. Wat Tam Khwan Meuang, Sawi, Chumphon: CUMZ 6015. Suan Somdet, Lang Suan, Chumphon: CUMZ 6017. Tam Khao Krieb, Lang Suan, Chumphon: CUMZ 6213 (3 specimens in ethanol).

###### Type locality.

Tam Phitsadan, Prathiew, Chumphon, Thailand, 10°43'26.6"N, 99°15'23.6"E.

###### Diagnosis.

This new species can be distinguished from *Discartemon plussensis*, *Discartemon leptoglyphus* and *Discartemon platymorphus* in having a nearly smooth shell surface, a shouldered last whorl, and five to seven apertural lamellae. *Discartemon afthonodontia* sp. n. differs from *Discartemon hypocrites* by having a nearly smooth shell surface and an aperture with two parietal, two palatal, one basal and two columella lamellae. The genitalia of *Discartemon afthonodontia* sp. n. differ from those of *Discartemon hypocrites* in lacking a penial appendix, in having the free oviduct long and slender, and in having the vas deferens passing straight through the penial sheath. They also differ from *Discartemon afthonodontia* sp. n. in having conical penial papillae, long and slender penial hooks, and in having the penial wall with thick reticulated folds, and the vaginal wall with a smooth surface. Compared with *Discartemon epipedis* sp. n., *Discartemon afthonodontia* sp. n. has more apertural lamellae, lacks a penial appendix and has the vas deferens passing straight through the penial sheath. They also differ from *Discartemon afthonodontia* sp. n. in having a penial wall with thick reticulated folds, and in having a very long and slender free oviduct.

###### Description.

**Shell.** Shell depressed-heliciform, white and translucent; whorls 6, spire conical to convex with distinct suture. Shell surface glossy, smooth with transverse ridges near the peristome and varices present only on early whorls. Embryonic shell large, about 2½ whorls, with a smooth surface; following whorls regularly coiled. Last whorl shouldered, sometimes angular with strong peripheral keel, regularly expanded, and two shallow and short longitudinal furrows present. Umbilicus widely open and deep. Aperture sub-quadrangular; peristome discontinuous, thickened, expanded and reflected. Aperture dentition with one strong parietal, one palatal, one basal, one large columellar and one small supracolumellar lamella; sometimes upper parietal and upper palatal lamellae present (Fig. 7A).

**Radula.** Each row consists of 35–39 teeth with formula (17-19)-1-(17-19). Central tooth small with pointed cusp. Lateral and marginal teeth undifferentiated, unicuspid and lanceolate. Latero-marginal teeth gradually reduce in size, with outermost teeth much smaller and shorter than inner teeth (Fig. 22D).

**Genital organs.** Atrium (at) short. Proximal penis (p) short with very short, stout penial appendix (pa). Distal penis slender (Fig. 13D, E). Penial sheath (ps) thin, extending about one and half times penis length; penial sheath retractor muscle (psr) very thin, originating at genital orifice wall and inserting distally on penial sheath (Fig. 13C). Vas deferens (vd) passes straight through penial sheath before entering into penis distally (Fig. 13D). Penial retractor muscle (pr) thin and very long, inserting at penis and vas deferens junction.

Internal wall of atrium with numerous atrial pores (Fig. 19A). Penial wall with scattered brown penial hooks, about 5 hooks/200 µm^2^ (Fig. 19B). Hooks located on conical penial papillae (pp) which are separated by thickened reticulated folds. Penial hooks small (<0.01 mm in length), expanded at base, tips pointed and curved towards genital orifice (Fig. 19C, D).

Vagina (v) short, about half of penis length. Gametolytic duct (gd) a long tube extending as far as albumin gland; gametolytic sac (gs) ovate. Free oviduct (fo) a very long and slender tube; oviduct (ov) enlarged and folded; prostate gland inconspicuous and bound to oviduct. Talon (ta) small and slender. Hermaphroditic duct (hd) bearing long seminal vesicle (sv) about one and half times longer than the length from talon to branching point of seminal vesicle (Fig. 13C).

Vaginal wall surface generally smooth (Fig. 19E).

###### Etymology.

The specific epithet “*afthonodontia*” is derived from the Greek “*afthonos*” meaning “plenty” and “*dontia*” meaning “teeth”.

###### Distribution.

This species is known from several limestone karsts in Chumphon and Prachuap Khirikhan Provinces, southern Thailand. This is a narrow range confined to the Isthmus of Kra area, from 9° to 11° N and 99° to 100° E.

###### Remarks.

Shell variations are detected across populations. In the Tam Khao Phlu (CUMZ 3581, 3666, 6214) and Khao Maeo (CUMZ 3589) populations, shells have a stronger peripheral keel, a subcircular aperture, and lack the upper parietal lamella (Fig. 7B). The specimens from Wat Tam Khao Marong (CUMZ 4219, 6014, 6211), Wat Tam Khwan Meuang (CUMZ 6015), and Suan Somdet (CUMZ 6017) exhibit a convex spire, and the upper parietal and upper palatal lamellae are sometimes absent. However, these five populations exhibit similar genitalia characters including the penial sculpture. Therefore, we consider them all conspecific.

##### 16. Discartemon epipedis

Siriboon & Panha sp. n.

http://zoobank.org/20AFF3F2-9EBE-4DF9-BFC1-B5551D93DDC4

http://species-id.net/wiki/Discartemon_epipedis

Figs 3A , 7C, D , 14A, B , 19F–I , 23 , Table 2

###### Type material.

Holotype CUMZ 6250 (Fig. 7C). Measurement: shell height 4.6 mm, shell width 8.7 mm, and with 6 whorls. Paratypes: CUMZ 6020 (15 shells), 6215 (5 specimens in ethanol; Figs 3A, 14A, B, 19F–I), NHMUK 20130678 (2 shells), and SMF (2 shells) from the type locality.

###### Type locality.

Gua Matu Madu, Gua Musang, Kelantan, Malaysia, 4°50'13.4"N, 101°56'56.3"E.

###### Diagnosis.

*Discartemon epipedis* sp. n. differs from *Discartemon plussensis* and *Discartemon leptoglyphus* in having a higher spire, a nearly smooth shell surface, a semi-ovate aperture, and four apertural lamellae. Compared with *Discartemon flavacandida* sp. n., *Discartemon epipedis* sp. n. has a smaller shell, lacks longitudinal furrows, has the last whorl rounded and regularly coiled, and has four apertural lamellae. The genitalia of *Discartemon epipedis* sp. n. differ from those of *Discartemon flavacandida* sp. n. in having a very short and swollen penial appendix, a long and enlarged vagina, short free oviduct, low conical penial hooks, penial papillae present, and in lacking vaginal pores. *Discartemon epipedis* sp. n. differs from *Discartemon roebeleni* in having a depressed-heliciform shell, a nearly smooth shell surface, and a semi-ovate aperture. The genitalia have a very short and swollen penial appendix, long and enlarged vagina, long and slender free oviduct, dark brown penial hooks located on conical penial papillae, and a vaginal wall with smooth surface.

###### Description.

**Shell.** Shell depressed-heliciform, white and translucent; whorls 6, spire only slightly convex with distinct suture. Shell surface glossy, nearly smooth with few transverse ridges near peristome; varices present. Embryonic shell large, about 2½ whorls. with a smooth surface; following whorls regularly coiled. Last whorl shouldered or rarely rounded, regularly expanded; umbilicus widely open and deep. Aperture semi-ovate; peristome discontinuous, thickened, expanded and reflected. Apertural dentition with a strong parietal lamella and one palatal, one basal and one columellar lamella (Fig. 7C).

**Genital organs.** Atrium (at) very short. Proximal penis (p) very short penial appendix (pa) swollen in middle, and distal penis slender. Penial sheath (ps) thin, extending about two-thirds of penis length; penial sheath retractor muscle (psr) very thin, originating at genital orifice wall and inserting distally on penial sheath (Fig. 14A). Vas deferens (vd) passes through about one-seventh of penial sheath length before entering into penis distally (Fig. 14B). Penial retractor muscle (pr) thin and very long, inserting at penis and vas deferens junction.

Internal wall of atrium generally smooth with sparse atrial pores (Fig. 19F); penial wall with scattered dark brown penial hooks, about 2 hooks/200 µm^2^ (Fig. 19G). Hooks located on conical penial papillae (pp) separated by thin reticulated folds. Penial hooks small (<0.03 mm in length), low conical, expanded at base, tips pointed (Fig. 17H).

Vagina (v) long, enlarged, about half of penis length. Gametolytic duct (gd) expanded at base and tapering to long and tube extending as far as albumin gland; gametolytic sac (gs) ovate. Free oviduct (fo) a long and narrow tube; oviduct (ov) enlarged and folded; prostate gland inconspicuous and bound to oviduct. Talon (ta) small, short and club shaped. Hermaphroditic duct (hd) bearing long seminal vesicle (sv) about three times longer than the length from talon to branching point of seminal vesicle (Fig. 14A).

Vaginal wall with smooth surface of strong recticulate vaginal folds (Fig. 17I).

###### Etymology.

The specific epithet “*epipedis*” is derived from the Greek “*epipedos*” meaning “flat” It refers to the flattened- or depressed-heliciform shell.

###### Distribution.

This species is known only from the type locality.

###### Remarks.

Apparently rare and only extensive searching yielded living animals.

##### 17. Discartemon flavacandida

Siriboon & Panha sp. n.

http://zoobank.org/D224A65B-6BD7-46A4-B7FE-45C7B143CA72

http://species-id.net/wiki/Discartemon_flavacandida

Figs 3B , 7E, F , 14C, D , 19J–N , 23 , Table 2

###### Type material.

Holotype CUMZ 6251 (Fig. 7E). Measurement: shell height 5.7 mm, shell width 11.7 mm, and with 6½ whorls. Paratypes: CUMZ 3574 (25 shells), 3576 (9 shells), 3579 (1 shell), 3580 (3 shells), 3675 (7 shells), 3676 (33 shells), 3677 (1 shell), 4214 (26 shells), 6006 (22 shells), 6216 (2 specimens in ethanol; Figs 3B, 14C, D, 19J–N), NHMUK 20130679 (2 shells), and SMF (2 shells) from the type locality.

###### Type locality.

Tam Phra Khayang, Kra Buri, Ranong, Thailand, 10°19'33.4"N, 98°45'54.7"E.

###### Diagnosis.

This new species is distinguished from *Discartemon plussensis*, *Discartemon leptoglyphus*, *Discartemon platymorphus*, *Discartemon roebeleni* and *Discartemon collingei* by having a larger shell with a smooth shell surface, a shouldered and slightly axially deflected last whorl, two longitudinal furrows and seven apertural lamellae. Its genitalia are distinctive in having a long but thick penial appendix.

###### Description.

**Shell.** Shell depressed-heliciform, white and translucent; whorls 6–6½, spire only slightly convex, with distinct suture. Shell surface glossy, smooth with thin growth lines.

Embryonic shell large, about 2½ whorls and with smooth surface; following whorls regularly coiled. Last whorl shouldered, slightly axially deflected, regularly expanded, and two short longitudinal furrows present. Umbilicus widely open and deep. Aperture semi-ovate; peristome discontinuous, expanded and reflected. Apertural dentition with strong parietal and small upper parietal lamellae separated at right angle, one small upper palatal, one palatal, one basal, one columellar, and one small supracolumellar lamella (Fig. 7E).

**Genitalia organs:** Atrium (at) long and slender. Penis (p) long; proximal penis slender; middle part enlarged with a long but thick penial appendix (pa) about half of penis length; distal penis long and slender (Fig. 14C). Penial sheath (ps) thin, extending about half of penis length; penial sheath retractor muscle (psr) very thin, originating at atrium and inserting distally on penial sheath. Vas deferens (vd) passes through about one-fifth of penial sheath length before entering into penis distally (Fig. 14D). Penial retractor muscle (pr) thin and very long, inserting at penis and vas deferens junction.

Internal wall of atrium generally smooth with numerous atrial pores (Fig. 19J). Penial wall with scattered light brown penial hooks, about 10 hooks/200 µm^2^ (Fig. 19K). Hooks located on penial wall. Penial hooks small (<0.03 mm in length), expanded at base, tips pointed and curved towards genital orifice (Fig. 19L, M).

Vagina (v) very short, about one-fourth of penis length. Gametolytic duct (gd) a long narrow tube extending as far as albumin gland; gametolytic sac (gs) ovate. Free oviduct (fo) very long, slender, proximal with equivalent diameter with vagina, and tapering to smaller tube distally. Oviduct (ov) folded and prostate gland inconspicuous. Talon (ta) small, and very short. Hermaphroditic duct (hd) bearing long seminal vesicle (sv) about one and half times longer than the length from talon to branching point of seminal vesicle (Fig. 14C).

Vaginal wall generally smooth, surface with numerous pores (Fig. 19N).

###### Etymology.

The specific epithet “*flavacandida*” is derived from the Latin “*flavus*” meaning “yellow” and “*candidus*” meaning “bright or transparent”.

###### Distribution.

This species seems to be restricted to limestone at the type locality.

###### Remarks.

Apparently rare and only extensive searching yielded living animals.

#### Group III: *Discartemon roebeleni*-group: Species with globose-heliciform shell.

##### 18. Discartemon lemyrei

(Morlet, 1883)

http://species-id.net/wiki/Discartemon_lemyrei

Figs 8A , 23

Streptaxis lemyrei Morlet, 1883: 104, 105, pl. 4, fig. 1. Type locality: Kampot, Cambodge. Tryon 1885: 67, pl. 16, figs 12, 13. Morlet 1889: 122. Gude 1903: 227.
Odontartemon (Discartemon) lemyrei – Kobelt 1906: 98, pl. 55, figs 13, 14. Kobelt 1910: 150.
Discartemon lemyrei – Benthem Jutting 1954: 79. Benthem Jutting 1959: 168. Richardson 1988: 183.

###### Material examined.

**Figure 8.**
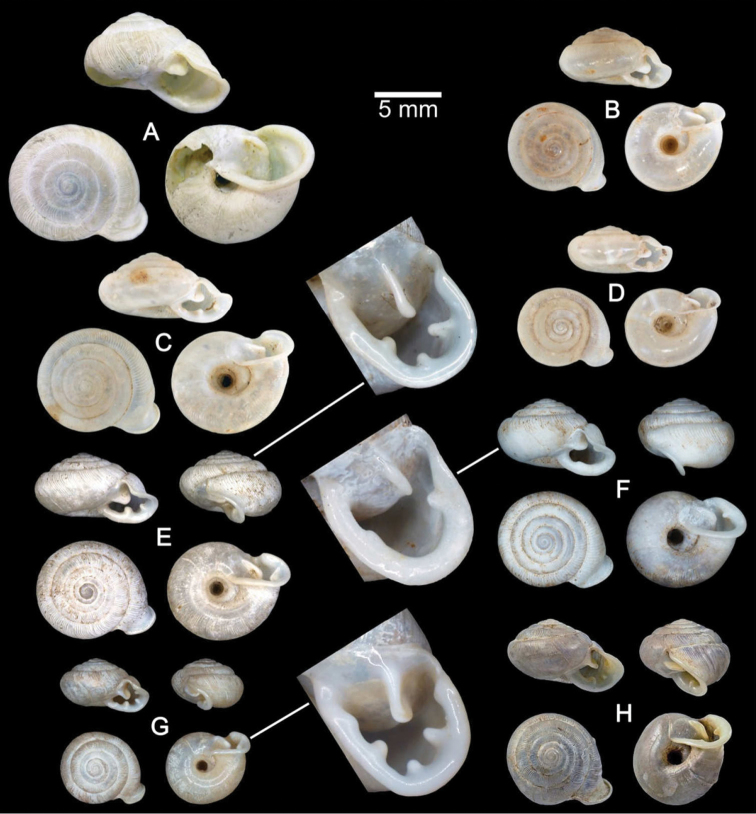
Shells of Group III: *Discartemon roebeleni*-group. **A** *Discartemon lemyrei* holotype MNHN **B–G** *Discartemon roebeleni* **B** lectotype SMF 108526 **C** holotype of forma *major* SMF 108531 **D** holotype of forma *minor* SMF 108533, **E** topotype CUMZ 3655 with apertural dentition **F** specimen CUMZ 3661, from Wat Suwankhuha, Phangnga with apertural dentition, and **G** specimen CUMZ 6256, from Ko Tarutao, Satun with apertural dentition. **H** *Discartemon collingei* syntype NHMUK 1937.7.9.20.

Holotype MNHN (Fig. 8A).

###### Remarks.

Shell globose-heliciform, spire elevated conical with distinct suture; following whorls regularly coiled. Shell surface with transverse ridges; last whorl rounded, regularly expanded; umbilicus unusually narrow. Aperture sub-quadrangular; peristome thickened, expanded and reflected. Apertural dentition with only one parietal lamella.

This species is very superficially similar to *Discartemon roebeleni* and *Discartemon collingei*, but has a larger shell with higher spire, unusually narrow umbilicus, and larger aperture with only a parietal lamella. In addition, the three species are allopatric, with *Discartemon lemyrei* occurring in Kampot and Panompen of Cambodia, while *Discartemon roebeleni* and *Discartemon collingei* occur in southern Thailand and peninsular Malaysia.

##### 19. Discartemon roebeleni

(Möllendorff, 1894)

http://species-id.net/wiki/Discartemon_roebeleni

Figs 3C , 8B–G , 15A–C , 20A–E , 22E , 23 , Table 3

Streptaxis roebeleni Möllendorff, 1894: 147, pl. 16, figs 3, 4. Type locality: Samui Island, Gulf of Siam. Gude 1903: 226. Gude 1920: 53. Laidlaw 1933: 233.
Odontartemon (Discartemon) roebeleni – Kobelt 1906: 99, pl. 54, figs 10, 11. Kobelt 1910: 150.
Discartemon roebeleni – Benthem Jutting 1954: 79, 81, fig. 3. Benthem Jutting 1959: 168. Zilch 1961: 82, pl. 5, fig. 4. Richardson 1988: 184. Maassen 2000: 88. Hemmen and Hemmen 2001: 42.

###### Material examined.

Lectotype of *Streptaxis roebeleni* SMF 108526 (Fig. 8B), and paralectotypes SMF 108527 (5 shells), 108528 (2 shells), 108529 (1 shell), 108530 (1 shell).

Holotype of forma *major* SMF 108531 (Fig. 8C), and paratype 108532 (2 shells). Holotype of forma *minor* SMF 108533 (Fig. 8D).

Topotypes from Samui, Thailand: NMW 1955.158.25255 (1 shell), and Nam Tok Hin Lad, Samui, Suratthani, Thailand, 9°31'15.3"N, 99°57'20.1"E: CUMZ 3655 (Fig. 8E), 4217, 6217 (52 specimens in ethanol; Figs 3C, 15A–C, 20A–E, 22E).

Topotypes from Samui, Thailand: NMW 1955.158.25255 (1 shell), and Nam Tok Hin Lad, Samui, Suratthani, Thailand, 9°31'15.3"N, 99°57'20.1"E: CUMZ 3655 (Fig. 8E), 4217, 6217 (52 specimens in ethanol; Figs 3C, 15A–C, 20A–E, 22E). Samui Island, Gulf of Siam [Thailand]: NHMW 36538 (1 shell), NHMW Rusnov R284 (1 shell), RMNH Fulton Coll. Reg. 177 (2 shells), ZMB 43127 (2 shells). Kow Tao Is. [=Ko Tao], Thailand: NMW 1955.158.25254 (7 shells). Ko Tao, Suratthani: CUMZ 3577. Ko Wuatalub, Ang Thong National Park, Suratthani: CUMZ 6022, 6218 (1 specimen in ethanol). Ko Mae Ko, Ang Thong National Park, Suratthani: CUMZ 6219 (1 specimen in ethanol). Ban Ta Khun, Suratthani: CUMZ 3590. Ratchaprapha reservior, Ban Ta Khun, Suratthani: CUMZ 6220 (4 specimens in ethanol). Khlong Saeng Wildlife Sanctuary, Ban Ta Khun, Suratthani: CUMZ 3652. Wat Khao Khok, Wiang Sa, Suratthani: CUMZ 3658. Wat Na San, Ban Na San, Suratthani: CUMZ 3578. Km 3, Khiri Rat Nikhom, Suratthani: CUMZ 6221 (1 specimen in ethanol). Wat Khao Phanom Wang, Suratthani: CUMZ 6222 (1 specimen in ethanol). Tam Hong, Khao Nan National Park, Nakhon Si Thammarat: CUMZ 4221. Tam Luang, Khao Nan National Park, Nakhon Si Thammarat: CUMZ 4231. Tam Phannara, Nakhon Si Thammarat: CUMZ 3667. Tam Khun Klung, Nopphitam, Nakhon Si Thammarat: CUMZ 6021. Khao Phrathong, Cha-uat, Nakhon Si Thammarat: CUMZ 3599. Wat Suwankhuha, Takua Thung, Phangnga: CUMZ 3661 (Fig. 8F), 6223 (14 specimens in ethanol). Sra Morakot, Krabi: CUMZ 6023, 6226 (7 specimens in ethanol). Ao Phra Nang, Krabi: CUMZ 3651. Khao Huai Hang, Huai Yot, Trang: CUMZ 3656. Tam Lay-Kao Krop, Huai Yot, Trang: CUMZ 3600. Botanic Garden, Trang: CUMZ 3663. Khao Pi-na, Na Yong, Trang: CUMZ 6024. Tam Sumno, Trang: CUMZ 6225 (1 specimen in ethanol). Khao Pu-Khao Ya National Park, Si Banphot, Phatthalung: CUMZ 3575, 3596. Tam Wang Thong, Phatthalung: CUMZ 3662, 6027, 6224 (6 specimens in ethanol). Wat Khaotupson, Phatthalung: CUMZ 3678. Khao Ok Thalu, Phatthalung: CUMZ 3595. Khao Chaison, Phatthalung: CUMZ 6028. Tam Tanan, Satun: CUMZ 6025. Tam Khantiphon, Satun: CUMZ 6026, 6227 (1 specimen in ethanol). Ko Buloan Pai, La Ngu, Satun: CUMZ 3591. Ko Tarutao, Satun: CUMZ 6228 (7 specimens in ethanol), 6256 (Fig. 8G). Ko Klang, Tarutao, Satun: CUMZ 6229 (9 specimens in ethanol). Khao Nui, Rattaphum, Songkhla: CUMZ 3598.

###### Description.

**Shell.** Shell globose-heliciform, white and translucent; whorls 6–6½, spire conical with distinct suture. Shell surface glossy, with transverse ridges that diminish below periphery; varices present. Embryonic shell large, about 2½ whorls, with a smooth surface; following whorls regularly coiled. Last whorl rounded, regularly expanded; umbilicus widely open and deep. Aperture sub-quadrangular; peristome discontinuous, thickened, expanded and reflected. Apertural dentition with one strong parietal, one palatal, one basal and one columellar lamella (Fig. 8B). Sometimes basal lamella absent (Fig. 8F), or upper palatal and supracolumellar lamellae present (Fig. 8G).

**Radula.** Each row consists of 21–33 teeth with formula (10-16)-1-(10-16). Central tooth very small and triangular with a pointed cusp. Lateral and marginal teeth undifferentiated, unicuspid and lanceolate. Latero-marginal teeth gradually reduce in size, with outermost teeth much smaller and shorter than inner teeth (Fig. 22E).

**Genitalia organs.** Atrium (at) long. Penis short and slender. Penial sheath (ps) extending entire penis length; penial sheath retractor muscle (psr) very thin, originating at genital orifice wall and inserting distally on penial sheath (Fig. 15A). Vas deferens (vd) passes through about one-seventh of penial sheath length before entering into penis distally (Fig. 15B). Penial retractor muscle (pr) thin and very long, inserting at penis and vas deferens junction.

Internal wall of atrium generally corrugated (Fig. 20A). Penial wall with scattered, transparent penial hooks, about 8 hooks/200 µm^2^. Hooks located on very short penial papilla (pp). Penial hooks small (<0.03 mm in length), short, expanded at base, tips pointed and curved towards genital orifice (Fig. 20C, D).

Vagina (v) short, about half of penis length. Gametolytic duct (gd) a long and slender tube extending as far as albumin gland; gametolytic sac (gs) ovate. Free oviduct (fo) short, about same length as vagina; oviduct (ov) folded; prostate gland inconspicuous and bound to oviduct. Talon (ta) small, very short. Hermaphroditic duct (hd) bearing extremely long seminal vesicle (sv) (Fig. 15C).

Vaginal wall generally with longitudinal vaginal folds (Fig. 20E).

**Table 3.** Shell measurements of *Discartemon* spp (*Discartemon roebeleni*-group). Specimen collections and catalogue numbers indicated in parentheses.

Species and locality and CUMZ nos	No. of specimens	Ranges, mean ± S.D. in mm of:			Number of whorls
		Shell height	Shell width	H/W ratio	
*Discartemon roebeleni* (Möllendorff, 1894)					
Ko Samui, Suratthani: (3655)	5	5.6–6.4 5.9±0.42	9.1–9.9 9.5±0.28	0.6–0.7 0.6±0.03	6–6½
Wat Khao Khok, Suratthani: (3658)	19	3.6–4.5 4.1±0.22	6.7–7.6 7.2±0.27	0.5–0.6 0.6±0.03	6–6½
Khao Nan N. P., Nakhon Si Thammarat: (4221)	10	4.5–5.3 4.8±0.27	7.1–9.1 8.1±0.61	0.6–0.6 0.6±0.03	6–6½
Wat Suwankhuha, Phangnga: (3661)	19	5.2–6.7 6.0±0.41	8.6–10.5 9.6±0.43	0.5–0.7 0.6±0.04	6–6½
Khao Pu-Khao Ya N. P., Phatthalung: (3596)	13	3.9–5.5 4.7±0.42	7.4–9.5 8.4±0.62	0.5–0.6 0.6±0.03	6–6½
Tam Wang Thong, Phatthalung: (3662, 6027)	15	4.4–5.6 4.9±0.31	7.0–8.0 7.6±0.29	0.6–0.7 0.6±0.03	6–6½
Khao Huai Hang, Trang: (3656)	9	4.8–5.6 5.2±0.34	8.2–9.5 9.1±0.52	0.5–0.6 0.6±0.02	6–6½
Botanic Garden, Trang: (3663)	9	5.1–6.4 5.6±0.38	8.0–10.3 8.9±0.81	0.6–0.7 0.6±0.04	6–6½
Sra Morakot, Krabi: (6023)	27	4.7–5.6 5.1±0.23	7.4–8.5 7.9±0.28	0.6–0.7 0.6±0.02	6–6½
Tam Tanan, Satun: (6025)	16	4.8–5.7 5.2±0.26	8.3–9.4 8.8±0.28	0.5–0.6 0.6±0.03	6–6½
Tam Khantiphon, Satun: (6026)	7	5.0–5.4 5.2±0.13	9.6–10.1 9.8±0.19	0.5–0.5 0.5±0.01	6–6½
*Discartemon kotanensis* sp. n.					
Ko Tan, Suratthani: (6230, 6252)	32	5.8–7.4 6.6±0.44	8.6–10.5 9.6±0.50	0.6–0.8 0.7±0.04	6–6½
*Discartemon megalostraka* sp. n.					
Nam Tok Tao Thong, Phangnga: (3657, 6031, 6253)	19	7.2–9.4 8.2±0.57	11.0–14.5 12.4±0.84	0.6–0.7 0.7±0.04	7–7½
Wat Tam Seua, Krabi: (6029)	6	6.5–7.3 6.9±0.3	10.7–11.6 11.2±0.34	0.6–0.6 0.6±0.02	7–7½
Ban Chong, Krabi: (6030)	10	7.7–9.4 8.5±0.52	12.0–12.9 12.6±0.32	0.6–0.7 0.7±0.04	7–7½
*Discartemon triancus* sp. n.					
Gunung Kilian, Perlis, Malaysia: (6234, 6254)	5	4.3–4.8 4.6±0.22	7.8–8.5 8.1±0.33	0.5–0.6 0.6±0.04	5½–6
*Discartemon conicus* sp. n.					
Gau Cerita, Langawi, Malaysia: (6033, 6255)	3	4.1–4.5 4.3±0.2	7.0–7.3 7.1±0.16	0.6–0.6 0.6±0.03	6

###### Distribution.

This species is found in limestone habitats and is common in southern Thailand. The geographic distribution records are in eight Provinces, ranging from 10°N to 6°N: Suratthani, Nakhon Si Thammarat, Krabi, Phangnga, Phatthalung, Trang, Satun, and Songkhla.

###### Remarks.

*Discartemon roebeleni* can be distinguished from *Discartemon collingei* by its rounded, regularly coiled last whorl and wider umbilicus. It differs from *Discartemon stenostomus* in the higher spire with transverse ridges that diminish below the periphery, a sub-quadrangular aperture, and in having a basal lamella.

##### 20. Discartemon collingei

(Sykes, 1902)

http://species-id.net/wiki/Discartemon_collingei

Figs 8H , 23

Streptaxis collingei Sykes, 1902: 22, 60, pl. 3, figs 8–10. Type locality: Kelantan, Malay Peninsula. Gude 1903: 214. Laidlaw 1933: 233.
Streptaxis (Discartemon) collingei – Möllendorff 1902: 136.
Odontartemon (Discartemon) collingei – Laidlaw 1929: 260.
Discartemon collingei – Benthem Jutting 1954: 79, 83, fig. 4. Benthem Jutting 1959: 168. Berry 1965: 28, 29. Richardson 1988: 182. Maassen 2001: 87. Marwoto 2008: 192.

###### Material examined.

Syntype NHMUK 1937.7.9.20 (1 shell; Fig. 8H). Kelantan, Malaysia: NMW 1955.158.25250 (2 shells). Kelantan, Malaysia: NHMW 40716 (1 shell).

###### Remarks.

Shell globose-heliciform, translucent, with a conical spire with a distinct suture. Shell surface with transverse ridges that diminish below periphery; varices present. Later whorls slightly axially deflected. Last whorl shouldered and regularly expanded; umbilicus wide and deep. Aperture sub-quadrangular; peristome thick, expanded and reflected. Apertural dentition with one parietal, one palatal, one basal and one columellar lamella (Fig. 8H).

*Discartemon collingei* is similar to *Discartemon lemyrei*, but the latter species has a larger shell with a higher spire, a shell surface with transverse ridges, the last whorl rounded and more inflated, a narrower umbilicus, and only one parietal lamella.

##### 21. Discartemon stenostomus

Benthem Jutting, 1954

http://species-id.net/wiki/Discartemon_stenostomus

Figs 9A, B , 23

Discartemon stenostomus – Benthem Jutting 1954: 83, 86, fig. 5. Type locality: Kaki Bukit, Perlis, Malaysia. Benthem Jutting 1959: 168. Berry 1965: 221–228, figs 1–3. Richardson 1988: 184. Maassen 2001: 88. Maassen 2003: 119. Marwoto 2008: 192.

###### Material examined.

**Figure 9.**
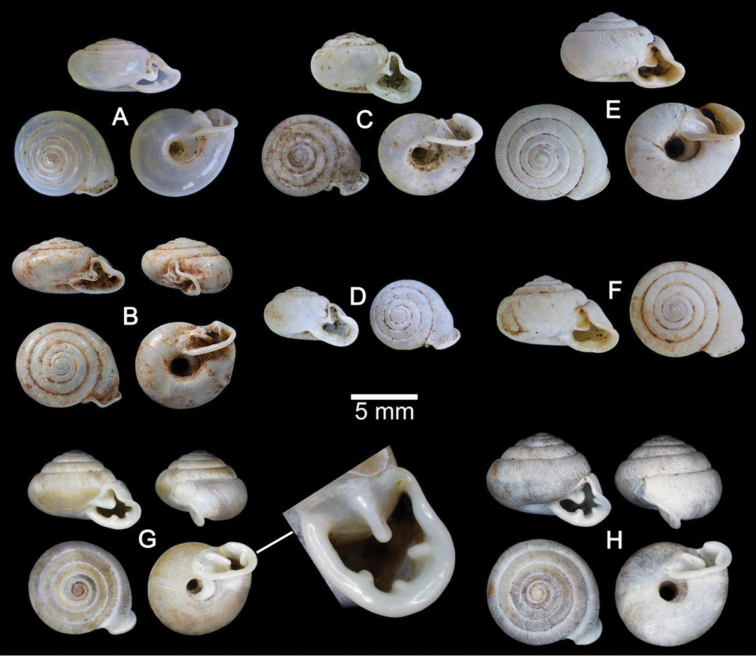
Shells of Group III: *Discartemon roebeleni*-group. **A, B** *Discartemon stenostomus* **A** holotype ZMA 3.54.024, and **B** paratype ZMA 3.54.025 **C, D** *Discartemon sangkarensis* **C** holotype ZMA 3.59.052, and **D** paratype ZMA 3.59.053 **E, F** *Discartemon vandermeermohri* **E** holotype ZMA 3.59.055, and **F** paratype ZMA 3.59.056. **G, H** *Discartemon kotanensis* sp. n. **G** holotype CUMZ 6252 with apertural dentition, and **H** paratype CUMZ 4220.

Holotype ZMA 3.54.024 (Fig. 9A). Paratypes ZMA 3.54.025 (8 shells), and NHMUK 1957-4-3.1-2 (2 shells). Kaki Bukit, Perlis: NMW 1955.158.25256 (10 shells).

###### Remarks.

Shell globose-heliciform, semi-transparent, spire only slightly convex and with a distinct suture. Shell surface glossy with thin transverse ridges at suture; following whorls regularly coiled. Last whorl rounded and regularly expanded; umbilicus widely open and deep. Aperture triangular; peristome thickened, expanded and reflected. Apertural dentition with one sinuous parietal, one palatal, one columellar and one supracolumellar lamella (Fig. 9A).

The genital anatomy was described by Berry (1965). Atrium and penis short with blunt penial appendix, penial sheath extending almost entire penis length, vas deferens passing through about one-fifth of penial sheath length. Internal wall of penis corrugated with cornified ridges, penial hooks absent, but with a large hollow “stylet” presumably protrudable from the tip of the everted penis. Vagina very short, proximal gametolytic duct enlarged, distally a long slender tube. Free oviduct short; talon small, club shaped; seminal vesicle about the same length from talon to branching point of seminal vesicle. Internal wall of vaginal elaborated with parallel vaginal folds.

This species resembles *Discartemon lemyrei* and *Discartemon collingei*, but differs in having thin transverse ridges near the suture, a triangular aperture, and apertural dentition with a sinuous parietal lamella, one palatal, one columellar and one supracolumellar lamella. In addition, *Discartemon lemyrei* has a larger shell, narrower umbilicus, and only one parietal lamella, while *Discartemon collingei* has a shouldered and slightly axially reflected last whorl, and apertural dentition with a straight parietal, one palatal, one basal and one columellar lamellae. A penial stylet has not yet been found in any other *Discartemon* species.

##### 22. Discartemon sangkarensis

Benthem Jutting, 1959

http://species-id.net/wiki/Discartemon_sangkarensis

Figs 9C, D , 23

Discartemon sangkarensis – Benthem Jutting 1959: 168–170, fig. 10. Type locality: Batu Sangkar, near Pajakombo, Padang Highlands, Indonesia. Richardson 1988: 184. Marwoto 2008: 191.

###### Material examined.

Holotype ZMA 3.59.052 (Fig. 9C). Paratypes: ZMA 3.59.053 (1 shell), ZMA 3.59.054 (4 shells), and ZMA 3.59.057 (9 shells).

###### Remarks.

Shell globose-heliciform, semi-transparent, with a conical spire and distinct suture. Shell surface glossy with fine transverse ridges; following whorls regularly coiled. Last whorl rounded and regularly expanded; umbilicus widely open and deep. Aperture triangular, with sinulus; peristome thickened, expanded and reflected. Apertural dentition of only one parietal lamella (Fig. 9C).

*Discartemon sangkarensis* differs from *Discartemon roebeleni* in having a more inflated last whorl, a triangular aperture, in having a sinulus, and the apertural dentition of only one parietal lamella. Compared with *Discartemon lemyrei* and *Discartemon vandermeermohri*, *Discartemon sangkarensis* differs in having a sinulus. Also, *Discartemon lemyrei* has a relatively larger shell and narrow umbilicus, while *Discartemon vandermeermohri* has a small basal lamella. *Discartemon sangkarensis* differs from *Discartemon collingei* in having a higher spire, the last whorl rounded, more inflated and regularly coiled, and in having a sinulus, a triangular aperture, and only one parietal lamella. Also, *Discartemon collingei* is slightly axially deflected.

##### 23. Discartemon vandermeermohri

Benthem Jutting, 1959

http://species-id.net/wiki/Discartemon_vandermeermohri

Figs 9E, F , 23

Discartemon vandermeermohri Benthem Jutting, 1959: 166–168, fig. 9. Type locality: Batu Sok, Pulu Weh, Indonesia. Richardson 1988: 185. Marwoto 2008: 191.

###### Material examined.

Holotype ZMA 3.59.055 (Fig. 9E). Paratypes ZMA 3.59.056 (2 shells) and RMNH Brandhorst Reg. 387 (1 shell).

###### Remarks.

Shell thickened, globose-heliciform, with a conical spire and distinct suture. Shell surface with strong transverse ridges; varices present; following whorls regularly coiled. Last whorl rounded and regularly expanded; umbilicus widely open and deep. Aperture triangular; peristome thickened, expanded and reflected. Apertural dentition of one parietal and one columellar lamella (Fig. 9E).

This species differs from *Discartemon lemyrei* in its smaller shell, widely open umbilicus, triangular aperture, and in having two apertural lamellae. *Discartemon vandermeermohri* is readily distinguished from *Discartemon roebeleni* and *Discartemon collingei* in its having a triangular aperture, and in lacking a basal lamella. Also, *Discartemon collingei* is slightly axially deflected.

##### 24. Discartemon kotanensis

Siriboon & Panha sp. n.

http://zoobank.org/4707CE37-6404-485D-9DCD-B58753958FB5

http://species-id.net/wiki/Discartemon_kotanensis

Figs 3D , 9G, H , 15D–F , 20F–J , 22F , 23 , Table 3

###### Type material.

Holotype CUMZ 6252 (Fig. 9G). Measurement: shell height 6.3 mm, shell width 9.2 mm, and with 6 whorls. Paratypes: CUMZ 4220 (27 shells), 6230 (15 specimens in ethanol; Figs 3D, 15D–F, 20F–J, 22F), NHMUK 20130680 (2 shells), and SMF (2 shells) from the type locality.

###### Type locality.

Ko Tan, Samui, Suratthani, Thailand, 9°22'18.9"N, 99°56'53.7"E.

###### Other material examined.

Nam Tok Tone Nga Chang, Had Yai, Songkhla: CUMZ 6231 (4 specimens in ethanol). Ban Chang Lang, Si-kao, Trang: CUMZ 6232 (2 specimens in ethanol).

###### Diagnosis.

Conchologically this new species superficially resembles *Discartemon roebeleni* and *Discartemon megalostraka* sp. n. It differs from *Discartemon roebeleni* in having a higher spire, a very long penis, a penial sheath extending fourth-fifths of the penis length, a smooth atrium wall with atrial pores, and a short seminal vesicle. It differs from *Discartemon megalostraka* sp. n. in having a smaller shell and apertural dentition of four lamellae, and a shorter free oviduct, vas deferens and seminal vesicle. *Discartemon kotanensis* sp. n. differs from *Discartemon stenostomus* and *Discartemon collingei* in having a higher spire, transverse ridges reaching the periphery, the last whorl rounded and regularly coiled, and apertural dentition of one straight parietal, one basal and one columellar lamella. Also, *Discartemon collingei* is slightly axially deflected.

###### Description.

**Shell.** Shell globose-heliciform, white and translucent; whorls 6–6½, spire elevated conical, with distinct suture. Shell surface glossy, with transverse ridges that diminish below periphery; varices present. Embryonic shell large, about 2½ whorls, with a smooth surface; following whorls regularly coiled. Last whorl rounded and regularly expanded; umbilicus widely open and deep. Aperture sub-quadrangular; peristome discontinuous, thickened, expanded and reflected. Apertural dentition of one strong parietal, one palatal, one basal and one columellar lamella (Fig. 9G).

**Radula.** Each row consists of 27–31 teeth with formula (13-15)-1-(13-15). The central tooth is very small and triangular with a pointed cusp. Lateral and marginal teeth are undifferentiated, unicuspid and lanceolate. Latero-marginal teeth gradually reduce in size, with outermost teeth much smaller and shorter than inner teeth (Fig. 22F).

**Genital organs.** Atrium (at) short. Penis (p) very long and slender. Penial sheath (ps) thin and extending about fourth-fifths of penis length, and penial sheath retractor muscle (psr) very thin, originating at genital orifice wall and inserting distally on penial sheath (Fig. 15D). Vas deferens (vd) passes a very short distance through penial sheath before entering into penis distally (Fig. 15E). Penial retractor muscle (pr) thin and very long, inserting at penis and vas deferens junction.

Internal wall of atrium generally smooth with atrial pores (Fig. 20F). Penial wall with dense and transparent penial hooks, about 20 hooks/200 µm^2^ (Fig. 20H). Hooks located on short penial papillae (pp). Penial hooks small (<0.03 mm in length), expanded at base, tips pointed and curved towards genital orifice (Fig. 20I).

Vagina (v) short, about one-fifth of penis length. Gametolytic duct (gd) a long tube extending as far as albumin gland; gametolytic sac (gs) ovate. Free oviduct (fo) short, of about same length as vagina. Oviduct (ov) slender and folded; prostate gland inconspicuous. Talon (ta) small, very short and club shaped. Hermaphroditic duct (hd) bearing long seminal vesicle (sv) about twice as long as the length from talon to branching point of seminal vesicle (Fig. 15F).

Vaginal wall generally with longitudinal vaginal folds (Fig. 20J).

###### Etymology.

The specific epithet is derived from the type locality of this new species, the Ko Tan, Ko Samui, Suratthani Province.

###### Distribution.

This species is known from the type locality and few limestone outcrops on the southern mainland.

###### Remarks.

Shells of this species from Samui, Suratthani were originally thought to belong to *Discartemon roebeleni*. After the genital system of *Discartemon kotanensis* sp. n. was examined and critically investigated, it was considered distinct enough to be a separate species.

##### 25. Discartemon megalostraka

Siriboon & Panha sp. n.

http://zoobank.org/05A48225-70FB-406F-857B-0B05C9D07754

http://species-id.net/wiki/Discartemon_megalostraka

**Figure 10.**
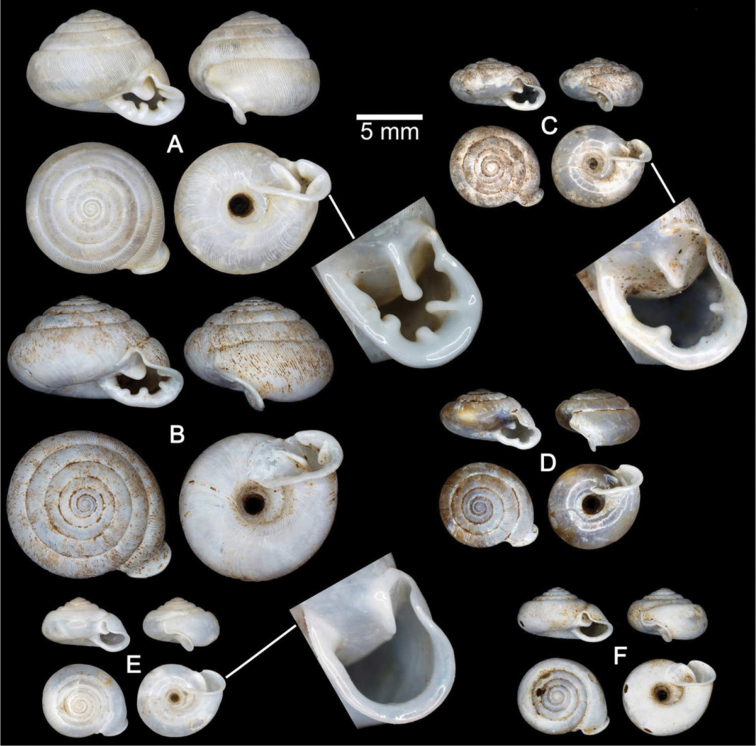
Shells of Group III: *Discartemon roebeleni*-group. **A, B** *Discartemon megalostraka* sp. n. **A** holotype CUMZ 6253 with apertural dentition, and **B** paratype CUMZ 3657 **C, D** *Discartemon triancus* sp. n. **C** holotype CUMZ 6254 with apertural dentition, and **D** paratype CUMZ 6032 **E, F** *Discartemon conicus* sp. n. **E** holotype CUMZ 6255 with apertural dentition, and **F** paratype CUMZ 6033.

Figs 3E , 10A, B , 16A–C , 21A–F , 22G , 23 , Table 3

###### Type material.

Holotype CUMZ 6253 (Fig. 10A). Measurement: shell height 8.0 mm, shell width 12.0 mm, and with 7 whorls. Paratypes: CUMZ 3657 (5 shells), 6031 (9 shells), 6233 (3 specimens in ethanol), NHMUK 20130681 (2 shells), and SMF (2 shells) from the type locality.

###### Type locality.

Nam Tok Tao Thong, Tub Pud, Phangnga, Thailand, 8°29'0.8"N, 98°35'4.8"E.

###### Other material examined.

Wat Tam Seua, Krabi: CUMZ 6029. Ban Chong, Krabi: CUMZ 6030. Wat Sathit Khirirom, Khirirat Nikhom, Suratthani: CUMZ 6234 (1 specimen in ethanol). Tam Wang Badan, Suratthani: CUMZ 6235 (2 specimens in ethanol).

###### Diagnosis.

This species differs from *Discartemon lemyrei* in its widely open umbilicus and apertural dentition of six lamellae. It differs from *Discartemon roebeleni* in having a much larger shell, higher spire, upper palatal and supracolumellar lamellae, a very long penis, penial sheath and free oviduct, shorter seminal vesicle, and in having atrial pores. *Discartemon megalostraka* sp. n. can be distinguished from *Discartemon stenostomus* in its larger shell, higher spire, transverse ridges, sub-quadrangular aperture, its straight parietal lamella, and in having upper palatal and basal lamellae.

###### Description.

**Shell.** Shell globose-heliciform, white and translucent; whorls 7–7½, spire elevated conical, with a distinct suture. Shell surface glossy with fine transverse ridges that diminish below periphery; varices present. Embryonic shell large, about 2½ whorls, with a smooth surface; following whorls regularly coiled. Last whorl rounded and regularly expanded; umbilicus widely open and deep. Aperture sub-quadrangular; peristome discontinuous, thickened, expanded and reflected. Apertural dentition of one strong parietal, one small upper palatal, one palatal, one basal, one columellar and one small supracolumellar lamella (Fig. 10A).

**Radula.** Each row consists of 19–21 teeth with formula (9-10)-1-(9-10). The central tooth is small and triangular with a pointed cusp. Lateral and marginal teeth are undifferentiated and large, unicuspid and lanceolate. Latero-marginal teeth gradually reduce in size, with outermost teeth much smaller and shorter than inner teeth (Fig. 22G).

**Genital organs.** Atrium (at) very short. Penis (p) very long and slender. Penial sheath (ps) thin, extending about third-fourths of penis length. Penial sheath retractor muscle (psr) very thin, originating at genital orifice wall and inserting distally on penial sheath (Fig. 16A). Vas deferens (vd) passes a very short distance through penial sheath before entering into penis distally (Fig. 16B). Penial retractor muscle (pr) thin and long, inserting at penis and vas deferens junction.

Internal wall of atrium with large atrial pores (Fig. 21A). Penial wall with scattered and transparent penial hooks, about 9 hooks/200 µm^2^ (Fig. 21B). Hooks located on penial wall. Penial hooks small (<0.02 mm in length), expanded at base, tips pointed and curved towards genital orifice (Fig. 21C–E).

Vagina (v) very short. Gametolytic duct (gd) a long and narrow tube extending as far as albumin gland; gametolytic sac (gs) ovate. Free oviduct (fo) extremely long, proximal part a straight cylindrical tube, distal part corrugated. Oviduct (ov) enlarged and folded; prostate gland inconspicuous. Talon (ta) small, very short and club shaped. Hermaphroditic duct (hd) bearing a long seminal vesicle (sv) about four times as long as the length from talon to branching point of seminal vesicle (Fig. 16C).

Vaginal wall with longitudinal vaginal folds (Fig. 21F).

###### Etymology.

The specific epithet “*megalostraka*” is derived from the Greek “*megalos*” meaning “big” and “*ostrako*” meaning “shell”.

###### Distribution.

This species is known from several limestone hills in southern Thailand, particularly in the western part of the southern mainland. The animals can be found at altitudes up to 20 meters amsl.

###### Remarks.

The genital system discriminates this new species from large individuals of *Discartemon roebeleni*, which is distributed throughout southern Thailand.

##### 26. Discartemon triancus

Siriboon & Panha sp. n.

http://zoobank.org/B067FBAC-0911-44A2-9949-D8C3DDDA2397

http://species-id.net/wiki/Discartemon_triancus

Figs 3F , 10C, D , 16D–F , 21G–L , 22H , 23 , Table 3

###### Type material.

Holotype CUMZ 6254 (Fig. 10C). Measurement: shell height 4.6 mm, shell width 7.3 mm, and with 6 whorls. Paratypes: CUMZ 6032 (2 shells), 6236 (6 specimens in ethanol), and NHMUK 20130682 (2 shells) from the type locality.

###### Type locality.

Gunung Kilian, Perlis, Malaysia, 6°34'8.0"N, 100°11'44.4"E.

###### Diagnosis.

This new species is superficially similar to *Discartemon roebeleni* and *Discartemon kotanensis* sp. n., but the distinguishing characters are the smaller shell, lower spire, angular last whorl, very long penis and free oviduct, short seminal vesicle, and penial hooks with elongated bases. *Discartemon triancus* sp. n. can be distinguished from *Discartemon megalostraka* sp. n. by having a smaller shell, lower spire, four apertural lamellae, a longer penis, short free oviduct, and slender penial hooks with elongated bases. *Discartemon triancus* sp. n. differs from *Discartemon conicus* sp. n. in having a lower spire with shallow suture, transverse ridges, in lacking a sinulus, and in having four apertural lamellae.

###### Description.

**Shell.** Shell globose-heliciform, white and translucent; whorls 5½–6, spire only slightly convex, with distinct suture. Shell surface glossy with transverse ridges that diminish below the periphery; varices present. Embryonic shell large, about 2½ whorls, with a smooth surface; following whorls regularly coiled. Last whorl angular, regularly expanded; umbilicus widely open and deep. Aperture subcircular; peristome discontinuous, thin and expanded. Apertural dentition of one parietal, one palatal, one small basal and one columellar lamella (Fig. 10C).

**Radula.** Each row consists of 27–43 teeth with formula (13-21)-1-(13-21). The central tooth is very small with pointed cusp. Lateral and marginal teeth are undifferentiated, unicuspid and lanceolate. Latero-marginal teeth gradually reduce in size, with outermost teeth much smaller and shorter than inner teeth (Fig. 22H).

**Genital organs.** Atrium (at) very short. Penis (p) extremely thin, long; becoming enlarged distally. Penial sheath (ps) thin, extending about half of penis length. Penial sheath retractor muscle very thin (psr), originating at atrium and inserting distally on penial sheath (Fig. 16D). Vas deferens (vd) passes a very short distance through penial sheath before entering into penis distally (Fig. 16E). Penial retractor muscle (pr) thin and very long, inserting at penis and vas deferens junction.

Internal wall of atrium generally smooth with pores (Fig. 21G). Penial wall with scattered and transparent penial hooks, about 11 hooks/200 µm^2^ (Fig. 21H). Hooks located on penial wall. Penial hooks small (<0.04 mm in length), short, with strongly elongated bases, tips pointed, and curved towards genital orifice (Fig. 21I–K).

Vagina (v) short. Gametolytic duct (gd) a long and slender tube extending as far as albumin gland; gametolytic sac (gs) ovate. Free oviduct (fo) with almost same diameter as vagina and about twice as long as vagina. Oviduct (ov) enlarged and folded; prostate gland inconspicuous. Talon (ta) small, short and slender. Hermaphroditic duct (hd) bearing a short seminal vesicle (sv) nearly equal to the length from talon to branching point of seminal vesicle (Fig. 16F).

Vaginal wall with longitudinal vaginal folds (Fig. 21L).

###### Etymology.

The specific epithet “*triancus*” is derived from the Latin “*triangulum*” meaning “triangle” and “*uncus*” meaning “hook”.

###### Distribution.

Known only from the type locality.

###### Remarks.

Material from Gunung Kilian, Perlis, Malaysia was firstly identified as *Discartemon roebeleni* (Möllendorff, 1894) by Benthem Jutting (1954), without any anatomical comparison. However, clear anatomical differences between this new species and *Discartemon roebeleni*, so it is considered a new species.

##### 27. Discartemon conicus

Siriboon & Panha sp. n.

http://zoobank.org/AE61F42F-34C8-4A3E-8825-1730EFAA23FA

http://species-id.net/wiki/Discartemon_conicus

Figs 10E, F , 23 , Table 3

###### Type material.

Holotype CUMZ 6255 (Fig. 10E). Measurement: shell height 4.5 mm, shell width 7.2 mm, and with 6 whorls. Paratypes: CUMZ 6033 (2 shells) from the type locality.

###### Type locality.

Gau Cerita, Langawi, Malaysia, 6°27'21.8"N, 99°49'29.8"E.

###### Diagnosis.

This species differs from *Discartemon roebeleni*, *Discartemon sangkarensis*, *Discartemon vandermeermohri* and *Discartemon kotanensis* sp. n., in having a smaller shell, higher spire, a nearly smooth shell surface, an angular last whorl, a sub-quadrangular aperture with a sinulus, and in having only one parietal lamella.

###### Description.

**Shell.** Shell globose-heliciform, white and translucent; whorls 6, spire elevated conical, with distinct suture. Shell surface glossy, smooth with thin transverse ridges near aperture; varices present. Embryonic shell large, about 2½ whorls, with a smooth surface; following whorls regularly coiled. Last whorl angular, inflated and regularly expanded. Umbilicus open and deep. Aperture sub-quadrangular with sinulus; peristome discontinuous, expanded and reflected. Apertural dentition of only one parietal lamella (Fig. 10E).

###### Distribution.

Known only from the type locality among limestone karsts up to 100 meters amsl, surrounded by mangrove forests on the northeast Langkawi Island coastline.

###### Remarks.

The new species is apparently rare and extensive searching yielded only three examples.

**Figure 11.**
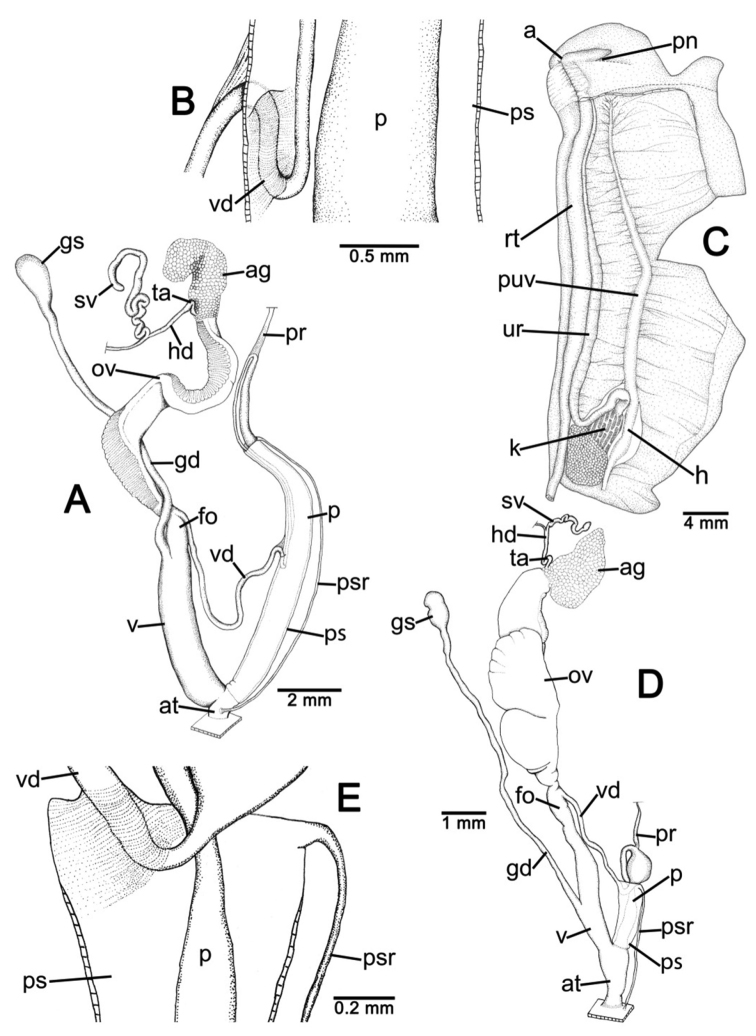
Genitalia and pallial complex. **A–C** *Discartemon discus* CUMZ 6257, from Vietnam **A** reproductive system **B** insertion of vas deferens into penial sheath, and **C** circular and excretory systems and mantle edge **D, E** *Discartemon nummus* CUMZ 6208, from Patthalung **D** reproductive system, and **E** insertion of vas deferens into penial sheath.

**Figure 12.**
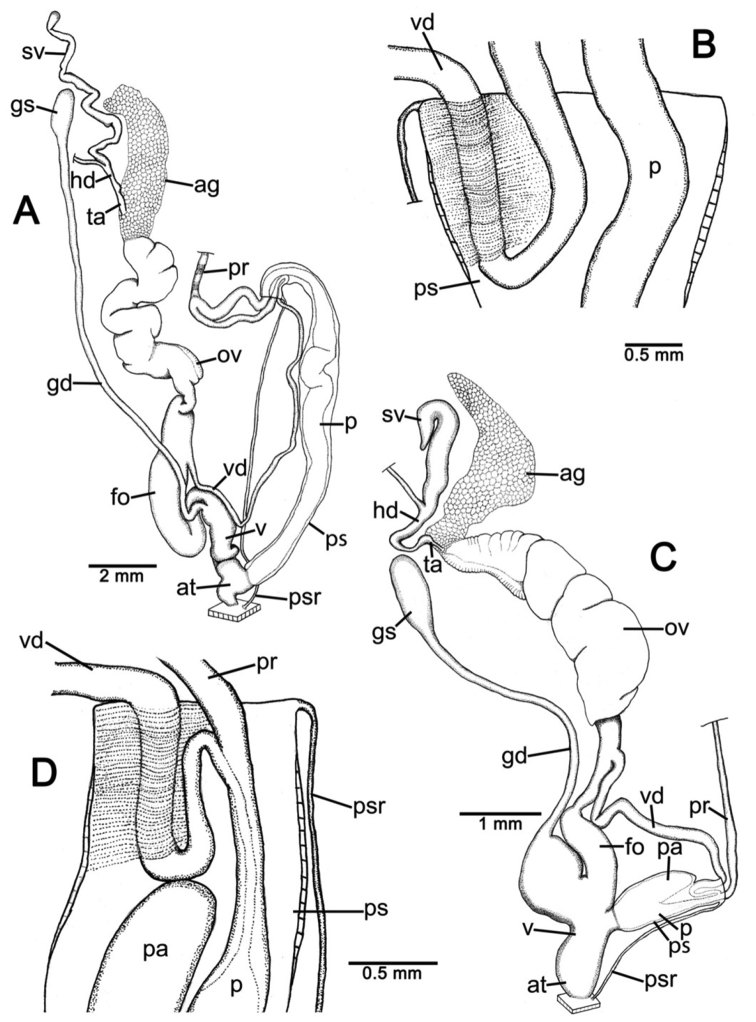
Genitalia. **A, B** *Discartemon discadentus* sp. n. paratype CUMZ 6209 **A** reproductive system, and **B** insertion of vas deferens into penial sheath **C, D** *Discartemon hypocrites* topotype CUMZ 6199 **C** reproductive system, and **D** insertion of vas deferens into penis sheath.

**Figure 13.**
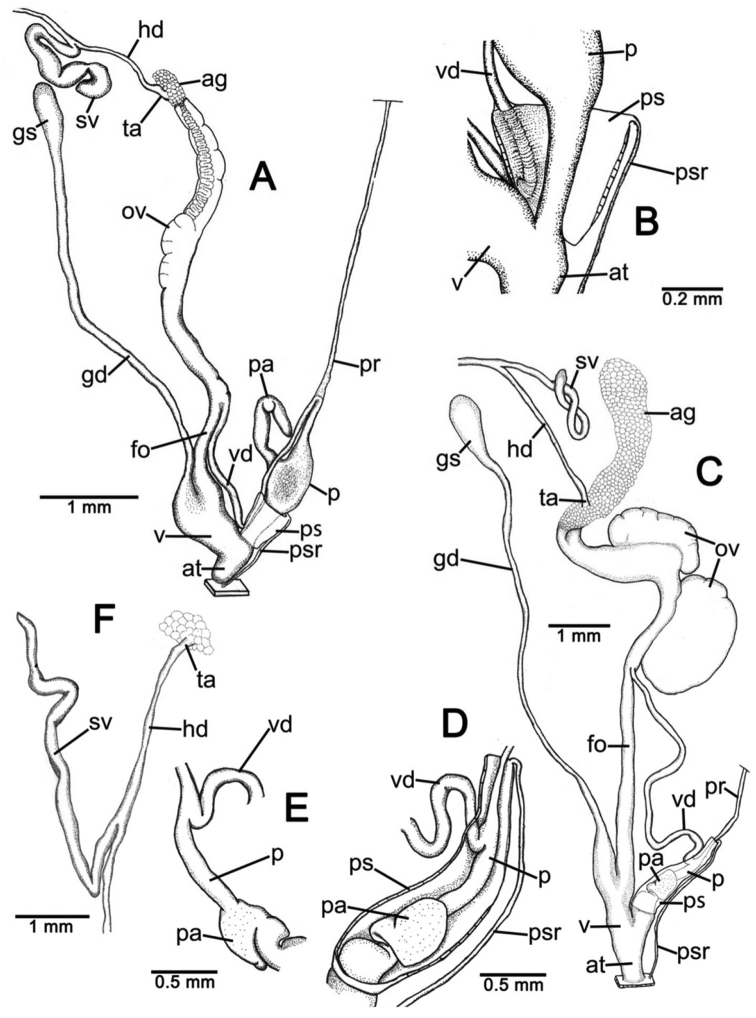
Genitalia. **A, B** *Discartemon leptoglyphus* CUMZ 6007, from Ipoh, Perak, Malaysia **A** reproductive system, and **B** insertion of vas deferens into penial sheath **C–F** *Discartemon afthonodontia* sp. n. paratype CUMZ 6210 **C** reproductive system **D** insertion of vas deferens into penial sheath **E** inflation of penis, and **F** details of hermaphroditic duct and seminal vesicle.

**Figure 14.**
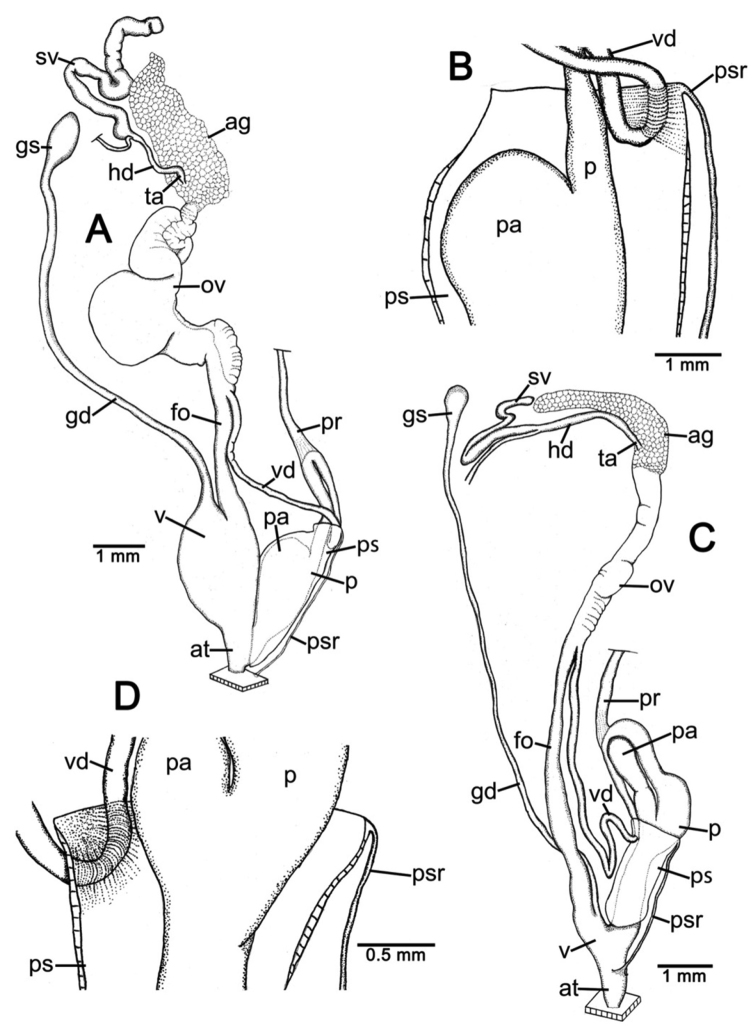
Genitalia. **A, B** *Discartemon epipedis* sp. n. paratype CUMZ 6215 **A** reproductive system, and **B** insertion of vas deferens into penis sheath **C, D** *Discartemon flavacandida* sp. n., paratype CUMZ 6216 **C** reproductive system, and **D** insertion of vas deferens into penis sheath.

**Figure 15.**
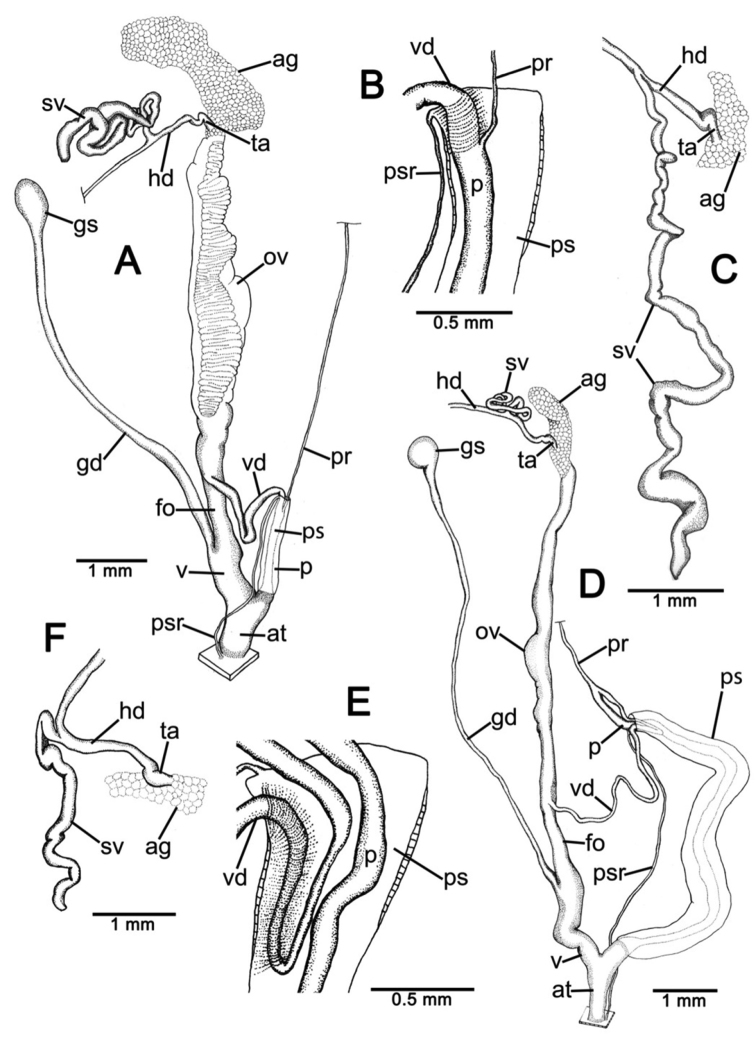
Genitalia. **A–C** *Discartemon roebeleni* topotype CUMZ 6217 **A** reproductive system **B** insertion of vas deferens into penial sheath, and **C** details of hermaphroditic duct and seminal vesicle **D–F** *Discartemon kotanensis* sp. n. paratype CUMZ 6230 **D** reproductive system, **E** insertion of vas deferens into penis sheath, and **F** details of hermaphroditic duct and seminal vesicle.

**Figure 16.**
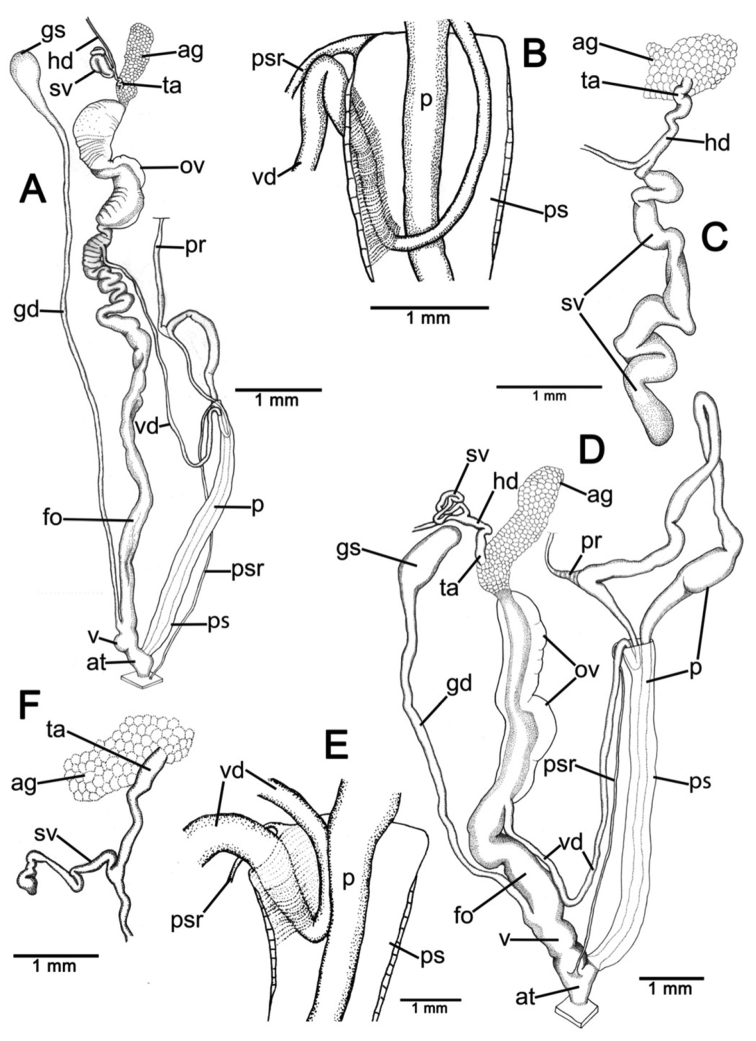
Genitalia. **A–C** *Discartemon megalostraka* sp. n., paratype CUMZ 6233 **A** reproductive system **B** insertion of vas deferens into penial sheath, and **C** details of hermaphroditic duct and seminal vesicle **D–F** *Discartemon triancus* sp. n., paratype CUMZ 6236 **D** reproductive system **E** insertion of vas deferens into penis sheath, and **F** details of hermaphroditic duct and seminal vesicle.

**Figure 17.**
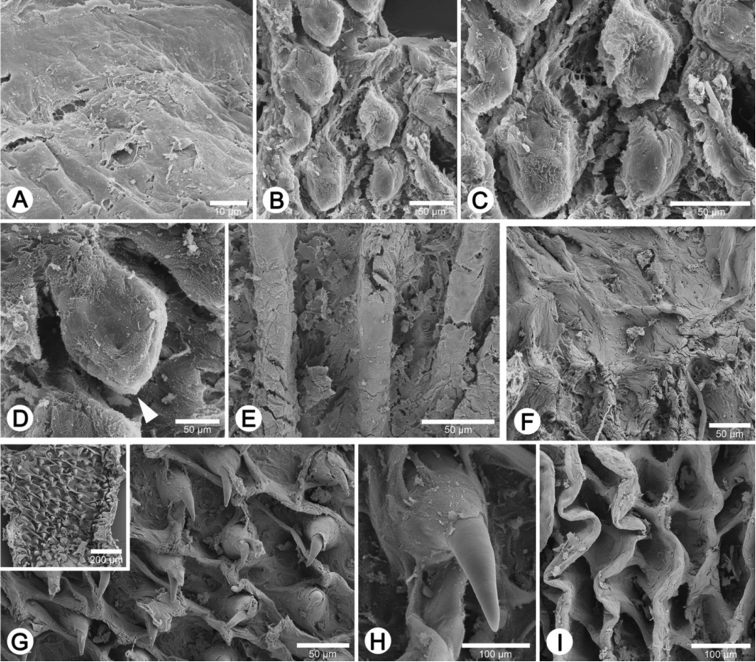
Internal sculpture of genitalia. **A–E** *Discartemon nummus* CUMZ 6208, from Patthalung **A** details of atrium surface **B** low magnification shows arrangement of penial hooks **C** high magnification of penial hooks **D** top view of penial hooks, white arrow indicate tip of hook, and **E** arrangement of vaginal folds **F–I** *Discartemon discadentus* sp. n., paratype CUMZ 6209 **F** details of atrium surface **G** high magnification of penial hooks with (inset) shows in low magnification **H** top view of penial hook, and **I** arrangement of reticulated vaginal folds.

**Figure 18.**
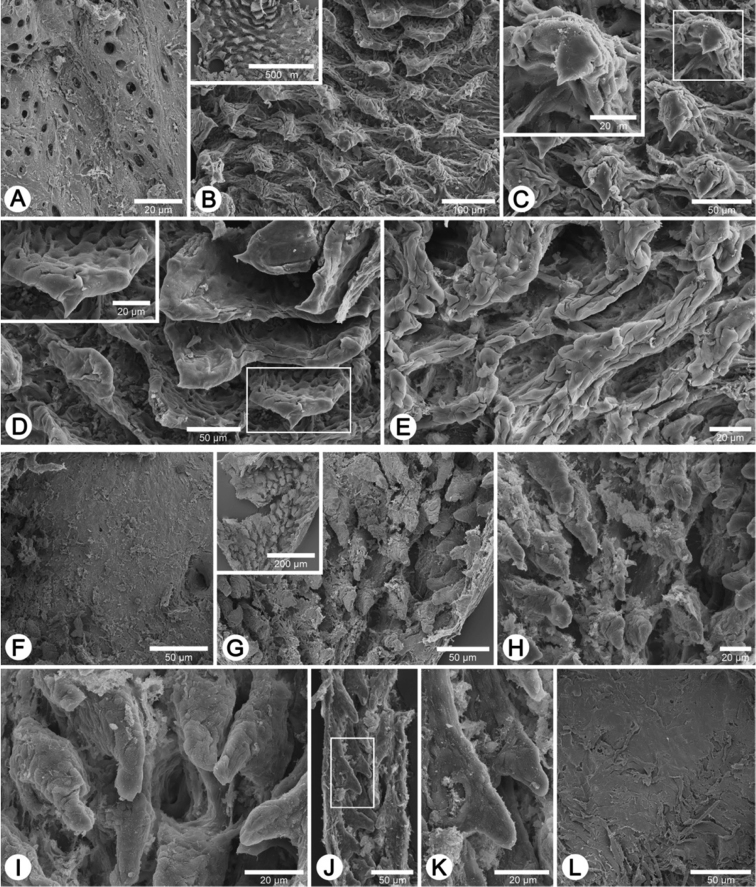
Internal sculpture of genitalia. **A–E** *Discartemon hypocrites*, topotype CUMZ 6199 **A** details of atrial pore on atrium surface **B** arrangement of penial hooks with high magnification, and (inset) shows in low magnification, **C** penial hooks with (inset) shows top view of the hook **D** penial hooks with (inset) shows lateral view of the hook, and **E** arrangement of vaginal folds **F–L** *Discartemon leptoglyphus* CUMZ 6007, from Ipoh, Perak, Malaysia **F** details of atrium surface **G** arrangement of penial hooks with (inset) low magnification **H** high magnification of penial hooks **I** top view of penial hook **J** low magnification shows lateral view of penial hooks **K** high magnification shows lateral view of penial hooks, and **L** details of vaginal surface.

**Figure 19.**
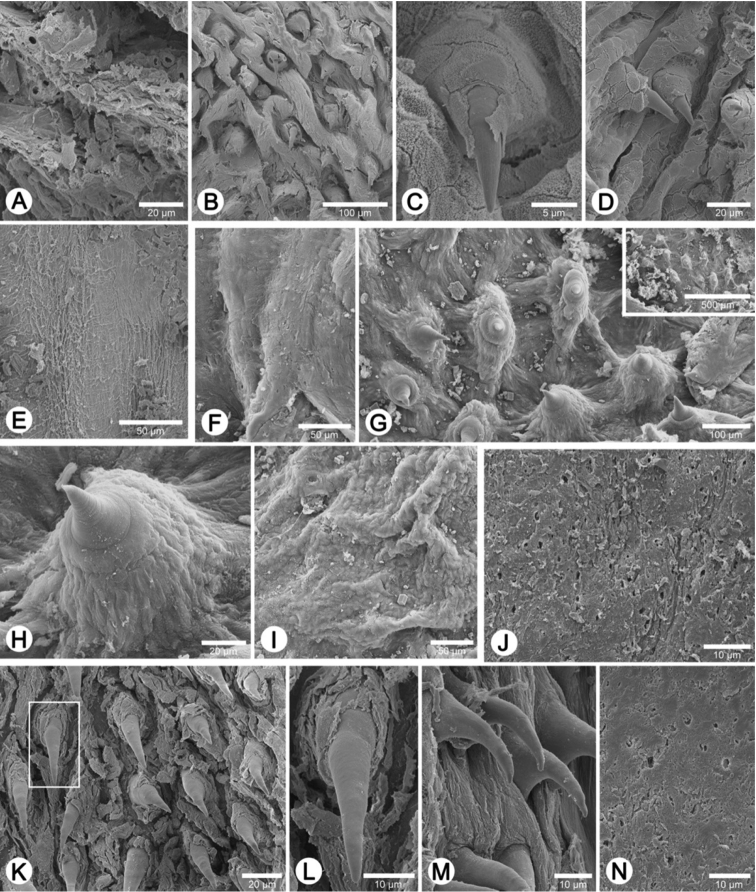
Internal sculpture of genitalia. **A–E** *Discartemon afthonodontia* sp. n. paratype CUMZ 6210 **A** details of atrial pore on the atrium surface **B** high magnification shows arrangement of penial hooks **C** top view of penial hook **D** lateral view of penial hook, and **E** details of vaginal surface **F–I** *Discartemon epipedis* sp. n. paratype CUMZ 6215 **F** details of the atrium surface **G** scattered arrangement of penial hooks with high magnification, and (inset) shows in low magnification **H** top view of penial hook, and **I** details of vaginal surface **J–N** *Discartemon flavacandida* sp. n. paratype CUMZ 6216 **J** details of atrial pore on the atrium surface **K** high magnification shows arrangement of penial hooks with top view of penial hook in white square **L** top view of penial hook (from white square in **K**) **M** lateral view of penial hook, and **N** details of vaginal surface.

**Figure 20.**
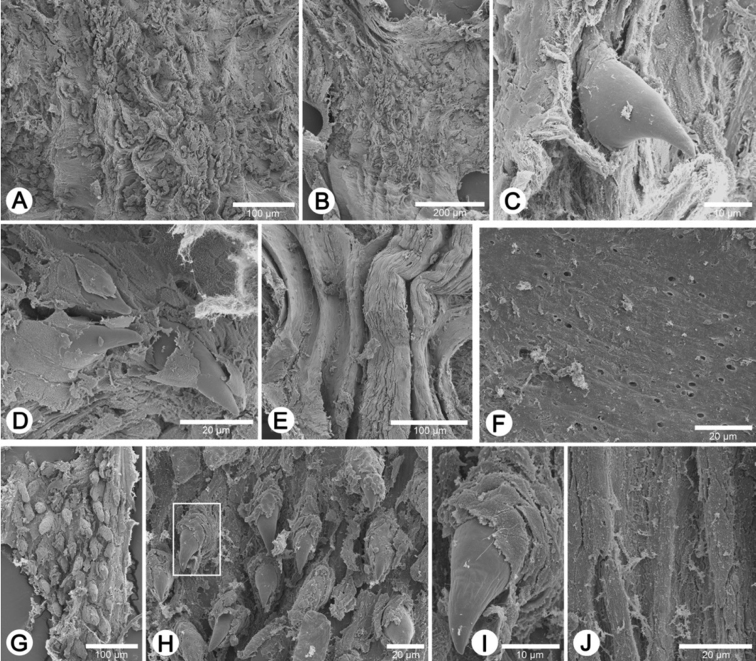
Internal sculpture of genitalia. **A–E** *Discartemon roebeleni* topotype CUMZ 6217 **A** details of atrium surface **B** low magnification shows arrangement of penial hooks **C** lateral view of penial hook **D** top view of penial hook, and **E** arrangement of vaginal folds **F–J** *Discartemon kotanensis* sp. n. paratype CUMZ 6230 **F** details of atrial pore on the atrium surface **G** low magnification shows arrangement of penial hooks **H** high magnification of penial hooks with top view of penial hook in white square **I** top view of penial hook (from white square in **H**), and **J** arrangement of vaginal folds.

**Figure 21.**
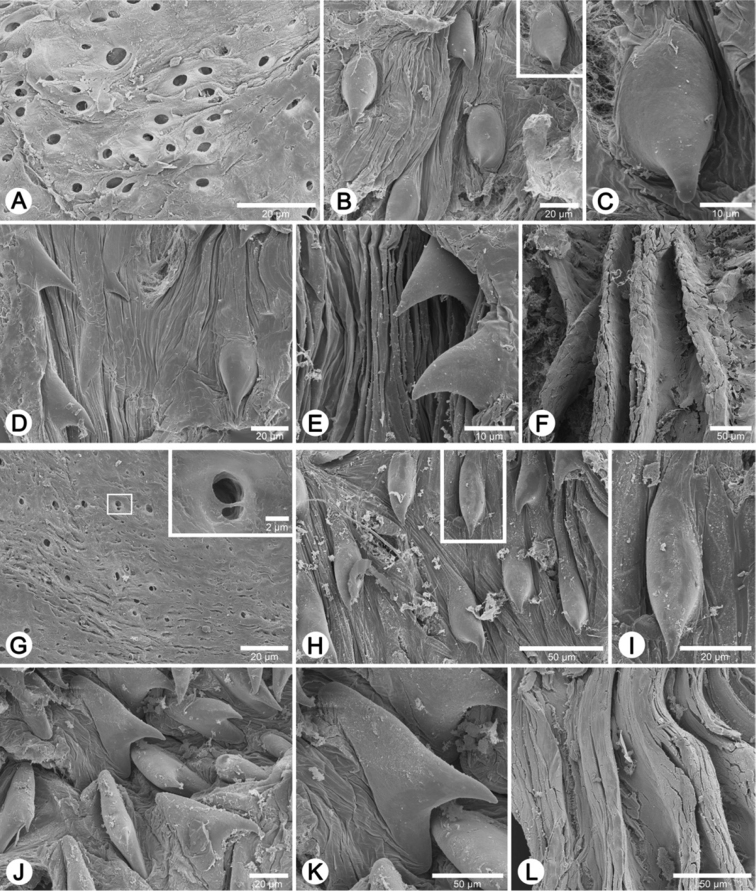
Internal sculpture of genitalia. **A–F** *Discartemon megalostraka* sp. n. paratype CUMZ 6233 **A** details of atrial pore on the atrium surface **B** high magnification of penial hooks with top view of penial hook in white square **C** top view of penial hook (from white square in **B**) **D** low magnification shows arrangement of penial hooks **E** lateral view of penial hook, and **F** arrangement of vaginal folds **G–L** *Discartemon triancus* sp. n. paratype CUMZ 6236 **G** details of atrial pore on the atrium surface with (inset) high magnification of atrial pore **H** high magnification of penial hooks with top view of penial hook in white square, **I** top view of penial hook (from white square in **H**) **J** arrangement of penial hooks **K** lateral view of penial hooks, and **L** arrangement of vaginal folds.

**Figure 22.**
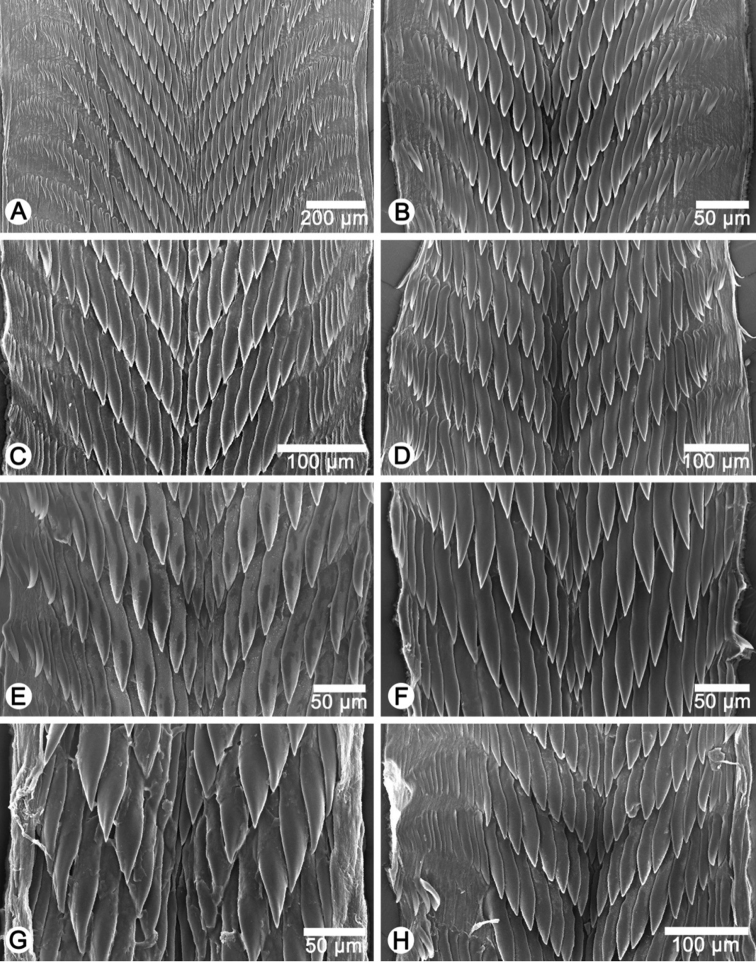
Radula morphology of **A** *Discartemon discus* CUMZ 6257, from Da Nang, Vietnam **B** *Discartemon nummus* CUMZ 6208, from Patthalung **C** *Discartemon hypocrites*, topotype CUMZ 6199 **D** *Discartemon afthonodontia* sp. n., paratype CUMZ 6210 **E** *Discartemon roebeleni*, topotype CUMZ 6217 **F** *Discartemon kotanensis* sp. n. paratype CUMZ 6230 **G** *Discartemon megalostraka* sp. n. paratype CUMZ 6233 **H** *Discartemon triancus* sp. n. paratype CUMZ 6236.

**Figure 23.**
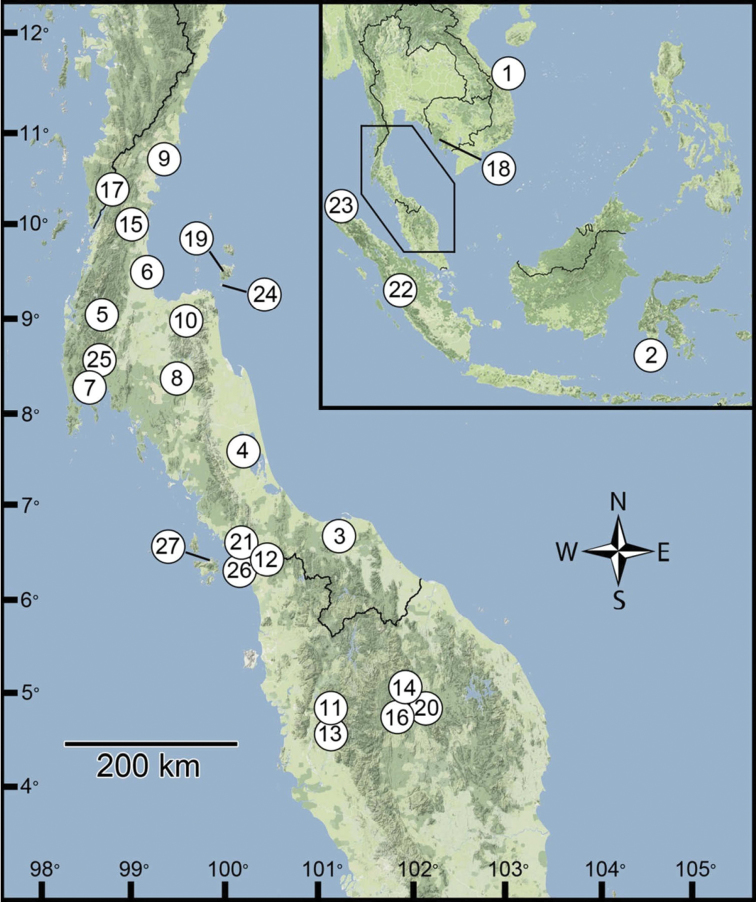
Approximate locations of the type locality of *Discartemon* species. (1) *Discartemon discus*, (2) *Discartemon planus*, (3) *Discartemon sykesi*, (4) *Discartemon nummus*, (5) *Discartemon khaosokensis*, (6) *Discartemon discadentus* sp. n., (7) *Discartemon discamaximus* sp. n., (8) *Discartemon circulus* sp. n., (9) *Discartemon deprima* sp. n., (10) *Discartemon expandus* sp. n., (11) *Discartemon plussensis*, (12) *Discartemon hypocrites*, (13) *Discartemon leptoglyphus*, (14) *Discartemon platymorphus*, (15) *Discartemon afthonodontia* sp. n., (16) *Discartemon epipedis* sp. n., (17) *Discartemon flavacandida* sp. n., (18) *Discartemon lemyrei*, (19) *Discartemon roebeleni*, (20) *Discartemon collingei*, (21) *Discartemon stenostomus*, (22) *Discartemon sangkarensis*, (23) *Discartemon vandermeermohri*, (24) *Discartemon kotanensis* sp. n., (25) *Discartemon megalostraka* sp. n., (26) *Discartemon triancus* sp. n., and (27) *Discartemon conicus* sp. n.

## Discussion

### Systematics

All species of *Discartemon* whose genital anatomy is known have a penial sheath through which the vas deferens passes for a short distance. This is typical of many streptaxid genera included in the subfamilies Streptaxinae Gray, 1860 and Gibbinae Steenberg, 1936 by Schileyko (2000). In contrast a penial appendix is a much less common feature and does not occur in any of these genera as treated by Schileyko (2000), although he did not cite Berry’s (1965) study of *Discartemon stenostomus*.

The species of *Discartemon* vary in whether an appendix is present and in other respects that correspond only approximately to the subdivision of the genus into three groups based on shell morphology. The groupings are as follows. Group I: *Discartemon discus*-group have a short to long, slender penis and transparent penial hooks. The genitalia of Group II: *Discartemon plussensis*-group have a short penis generally with a penial appendix, and transparent to brown penial hooks. A stout seminal vesicle may be present, and the gametolytic duct is usually enlarged and stout at the base. Group III: *Discartemon roebeleni*-group have a short to very long penis, sometimes with a blunt appendix, penial hooks are transparent, short, and expanded at the base. In one case *Discartemon stenostomus*, no penial hooks are present but a stylet is. The latter is the only *Discartemon* species whose genital anatomy was known prior to this study. Although its shell is not unusual for the genus, the species is apparently atypical in having a hollow stylet in the apex of the penis, which was not noted in other species. The function of the appendix and stylet are not known.

Genital anatomy does appear to be useful in the characterization and diagnosis of species-group taxa, however, particularly in the *Discartemon plussensis*-group.

### Biogeography

The distributional range of this genus is more extensive than previously known. Twenty-two of the species recorded in this study occur in the area from Isthmus of Kra to the western part of Malaysia including the Lankawi Islands. The other five species can be found in other limestone areas; Cambodia, central Vietnam, Sumatra and Sulawesi.

The genus apparently usually occurs in limestone habitats such as karst islands, isolated limestone hills and limestone mountains. Many Southeast Asian mollusks are restricted to such areas which are often threatened (Tweedie 1961; Clements et al. 2006). Furthermore many of the species here have very restricted distributions. Most are allopatric and a number appear to be endemic to single limestone hills. Others range throughout a limestone complex.

Three syntopic occurrences where one of the few common and widespread species, *Discartemon roebeleni*, occurs near restricted endemics were observed in this study. These were *Discartemon nummus*, *Discartemon circulus* sp. n., and *Discartemon deprima* sp. n. at Khao Ok Thalu, Phatthalung, Khao Pu-Khao Ya National Park, Patthalung, and Tam Phannara, Nakhon Si Thammarat respectively, all in southern Thailand.

## Supplementary Material

XML Treatment for Discartemon

XML Treatment for Discartemon discus

XML Treatment for Discartemon planus

XML Treatment for Discartemon sykesi

XML Treatment for Discartemon nummus

XML Treatment for Discartemon khaosokensis

XML Treatment for Discartemon discadentus

XML Treatment for Discartemon discamaximus

XML Treatment for Discartemon circulus

XML Treatment for Discartemon deprima

XML Treatment for Discartemon expandus

XML Treatment for Discartemon plussensis

XML Treatment for Discartemon hypocrites

XML Treatment for Discartemon leptoglyphus

XML Treatment for Discartemon platymorphus

XML Treatment for Discartemon afthonodontia

XML Treatment for Discartemon epipedis

XML Treatment for Discartemon flavacandida

XML Treatment for Discartemon lemyrei

XML Treatment for Discartemon roebeleni

XML Treatment for Discartemon collingei

XML Treatment for Discartemon stenostomus

XML Treatment for Discartemon sangkarensis

XML Treatment for Discartemon vandermeermohri

XML Treatment for Discartemon kotanensis

XML Treatment for Discartemon megalostraka

XML Treatment for Discartemon triancus

XML Treatment for Discartemon conicus
